# An anticodon-sensing T-boxzyme generates the elongator nonproteinogenic aminoacyl-tRNA *in situ* of a custom-made translation system for incorporation

**DOI:** 10.1093/nar/gkae151

**Published:** 2024-03-13

**Authors:** Wei Lu, Naohiro Terasaka, Yuriko Sakaguchi, Takeo Suzuki, Tsutomu Suzuki, Hiroaki Suga

**Affiliations:** Department of Chemistry, Graduate School of Science, The University of Tokyo, 7-3-1 Hongo, Bunkyo-ku, Tokyo 113-0033, Japan; Department of Chemistry, Graduate School of Science, The University of Tokyo, 7-3-1 Hongo, Bunkyo-ku, Tokyo 113-0033, Japan; Earth-Life Science Institute, Tokyo Institute of Technology, 2-12-1 Ookayama, Meguro-ku, Tokyo 152-8550, Japan; Department of Chemistry and Biotechnology, Graduate School of Engineering, The University of Tokyo, 7-3-1 Hongo, Bunkyo-ku, Tokyo 113-0033, Japan; Department of Chemistry and Biotechnology, Graduate School of Engineering, The University of Tokyo, 7-3-1 Hongo, Bunkyo-ku, Tokyo 113-0033, Japan; Department of Medical Biochemistry, Graduate School of Medicine, University of the Ryukyus, 207 Uehara, Nishihara, Okinawa 903-0125, Japan; Department of Chemistry and Biotechnology, Graduate School of Engineering, The University of Tokyo, 7-3-1 Hongo, Bunkyo-ku, Tokyo 113-0033, Japan; Department of Chemistry, Graduate School of Science, The University of Tokyo, 7-3-1 Hongo, Bunkyo-ku, Tokyo 113-0033, Japan

## Abstract

In the hypothetical RNA world, ribozymes could have acted as modern aminoacyl-tRNA synthetases (ARSs) to charge tRNAs, thus giving rise to the peptide synthesis along with the evolution of a primitive translation apparatus. We previously reported a T-boxzyme, Tx2.1, which selectively charges initiator tRNA with *N*-biotinyl-phenylalanine (BioPhe) *in situ* in a Flexible In-vitro Translation (FIT) system to produce BioPhe-initiating peptides. Here, we performed *in vitro* selection of elongation-capable T-boxzymes (elT-boxzymes), using para-azido-l-phenylalanine (Phe^AZ^) as an acyl-donor. We implemented a new strategy to enrich elT-boxzyme-tRNA conjugates that self-aminoacylated on the 3′-terminus selectively. One of them, elT32, can charge Phe^AZ^ onto tRNA *in trans* in response to its cognate anticodon. Further evolution of elT32 resulted in elT49, with enhanced aminoacylation activity. We have demonstrated the translation of a Phe^AZ^-containing peptide in an elT-boxzyme-integrated FIT system, revealing that elT-boxzymes are able to generate the Phe^AZ^-tRNA in response to the cognate anticodon *in situ* of a custom-made translation system. This study, together with Tx2.1, illustrates a scenario where a series of ribozymes could have overseen aminoacylation and co-evolved with a primitive RNA-based translation system.

## Graphical Abstract

**Graphical Abstract:**
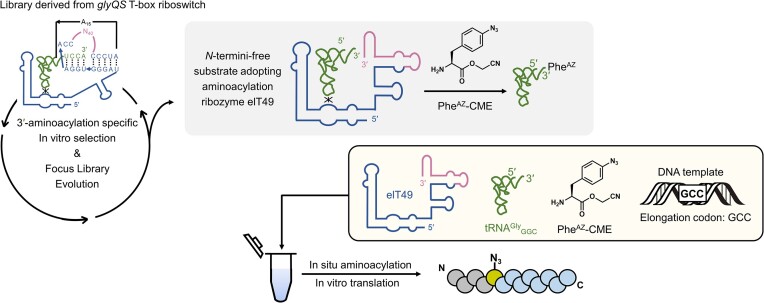

## Introduction

The rise of templated peptide synthesis in the ‘RNA world’ could be critical for the transition to the modern theatre of proteins (1–4). As the ribosomal peptidyl transfer center has been revealed to be a ribozyme (5), it suggests the possibility of an RNA-based primitive translation system in the early stage of life (1,6–8). While aminoacyl-tRNAs are essential intermediates for ribosomal protein synthesis in all living cells, this process is soloed by a family of protein enzymes, aminoacyl-tRNA synthetases (ARSs), in modern organisms (9). However, prior to the evolution of a sophisticated ‘proteins-RNAs-based’ translation system, ribozymes could have played a critical role in tRNA aminoacylation.

With the help of powerful *in vitro* selection, the first aminoacylation ribozyme was reported in 1995 to charge its own 3′-end with phenylalanine adenylate (Phe-AMP) (10). Later, many attempts have been made to develop aminoacylation ribozymes that transfer acyl from amino acid-AMPs (11–15), CoA-thioesters (16), biotinyl aminoacyl oxazolones (17,18) or 5′-aminoacyl phosphate oligonucleotide (19). Even though these ribozymes have the ability to aminoacylate RNA molecules, they were not catalysts for tRNA 3′-aminoacylation.

In the 2000s, our group generated a series of acyl-transfer ribozymes that charge an aminoacyl group on the 3′-terminus of tRNAs *in trans* for the first time (20–24). These outcomes later allowed us to develop a series of *trans*-acting tRNA-acylating ribozymes, referred to as flexizymes (25–28). The flexizymes recognize various aminoacyl-donors with ester or thioester leaving group and, at the same time, accept any tRNA-like molecules bearing 5′-NCCA-3′ at its 3′-end. This ‘flexibility’ of the substrate recognitions by the flexizymes makes them a versatile and convenient tool for preparing various kinds of acyl-tRNAs, whose acyl molecules could be not only nonproteinogenic α-amino acids (29–36) but also β/γ-amino acids (37–41), hydroxy acids (42,43), thio acids (44,45), amino (carbothio) acid (46), aminoxy and hydrazine acids (47). Nevertheless, the discovery of the flexizymes hints that such tRNA-aminoacylation ribozymes could have evolved in an ‘RNA world’.

Inspired by the flexizymes, we questioned the possibility of ribozymes resembling modern ARSs that could conjugate a specific amino acid to a cognate tRNA. In 2020, we employed the scaffold of naturally occurring tRNA-sensing *glyQS* T-box riboswitch from *Bacillus subtilis* (48,49) and developed aminoacylation ribozymes, T-boxzymes, by *in vitro* selection. One of the T-boxzymes, named Tx2.1, recognizes both its cognate tRNA and its substrate *N*-biotinyl-l-phenylalanine cyanomethyl ester (BioPhe-CME) (50), similar to natural protein ARSs. Therefore, Tx2.1 is capable of aminoacylating tRNA with BioPhe *in situ*, thus enabling the synthesis of BioPhe-initiating peptide in the custom-made *in vitro* translation (Flexible In-vitro Translation, FIT) system (51). On the other hand, we found that the *N*-biotinyl group of BioPhe-CME is involved in aminoacyl-donor recognition by Tx2.1. Therefore, Tx2.1 only applies to expressing peptides bearing biotin at the *N*-terminus.

Here, we aimed to develop new tRNA-sensing aminoacylation T-boxzymes that adopt *N*-termini-free substrates. During our first attempt, we encountered isolation of internal 2′-aminoacylation T-boxzymes (inT-boxzymes). To avoid such unwanted outcome, we devised a new *in vitro* selection method, referred to as biotin-based tRNA extension (b-tREX), to selectively isolate those specific to the tRNA 3′-aminoacylation, using para-azido-l-phenylalanine cyanomethyl ester (Phe^AZ^-CME) as a substrate. This b-tREX successfully yielded self-aminoacylating ribozymes on tRNA 3′-terminal hydroxyl group, and among them, we identified several *trans*-aminoacylation ribozymes, referred to as elongation-capable T-boxzymes (elT-boxzymes). One of them, elT32, was chosen for its selectivity towards tRNA anticodon and further evolved to promote its activity, resulting in elT49. These elT-boxzymes were further demonstrated to aminoacylate elongator tRNA *in situ* of the FIT system and produce a Phe^AZ^-containing peptide.

## Materials and methods

### Chemical synthesis of amino acid substrates

The amino acid substrates used in this study were synthesized from *N*-Boc-protected amino acids according to the methods described below. Phe^AZ^-CME, Phe-CME, d-Phe-CME, Tyr-CME and Trp-CME were prepared using the protocol described in (26). The preparation of Phe^AZ^-OH, Phe^AZ^-DBE, Gly-CME and Ala-CME followed the same procedure, while Phe^AZ^-OH omitted the step of carboxyl acid activation. BioPhe-CME was synthesized according to the method as described in (52), OH-Phe-CME as in (42), NMePhe-CME as in (33), AcPhe-CME and AcPhe^AZ^-CME as in (35).

### Preparation of DNA templates encoding RNA libraries

DNA template design and preparation were done by two-step PCR following the procedure described in (50). In the first step of DNA extension, designed oligos (oligo 2 and 3 for Library01, oligo 29 and 30 for Library02, oligo 29 and 31 for Library03) were added to 500-μl-scale PCR mix containing Taq polymerase Hot Start Version (R007A, TaKaRa), followed by thermocycle of 95°C 30 s, and five cycles of 55°C 30 s and 72°C 1 min. In the second step of PCR, the total amount of the extension product was added to a 5-ml-scale PCR mix containing Taq polymerase Hot Start Version and further extended to full-length with oligo 1 and 4 for six thermocycles of 95°C 30 s, 55°C 30 s and 72°C 1 min. Then, the DNA library was extracted by a phenol–chloroform mixture and precipitated with ethanol.

### *In vitro* transcription and purification of RNA

*In vitro* transcription with T7 polymerase was used to prepare the corresponding RNA. After transcription, the resulting RNA was purified by denaturing PAGE containing 8 M urea and 1× TBE. The absorbance at 260 nm determined the concentration of purified RNA.

### Aminoacylation

eFx-catalyzed aminoacylation was performed as previously described (35). The conditions of T-boxzyme self-aminoacylation were as follows: 3.33 μM RNA was heated at 95°C for 2 min and cooled on ice for 3 min. Then, this RNA solution was mixed with 1.667 volume of 2× reaction buffer (100 mM HEPES–KOH (pH = 7.5), 1M KCl, 20 mM MgCl_2_) and 25 mM Phe^AZ^-CME or corresponding amino acid substrates in DMSO. The mixture was incubated on ice for 2 h and stopped by adding four volumes of ice-cold 0.3 M sodium acetate (pH = 5.2). For *trans*-aminoacylation, 3.33 μM tRNA and 6.67 μM of selected ribozymes were mixed, then followed the previously described method to perform aminoacylation.

The 2× reaction buffer for self-aminoacylation used in elT32 evolution and *trans-*aminoacylation of resulting evolved elT-boxzymes was changed to (100 mM HEPES–KOH (pH = 7.5), 200 mM KOAc, 20 mM Mg(OAc)_2_) to simulate the condition used in *in vitro* translation.

### Copper-free click

Aminoacylated RNA was first precipitated by ethanol and resuspended in Tx reaction buffer (50 mM HEPES–KOH (pH = 7.5), 500 mM KCl, 5 mM EDTA) to have 1.25 μM RNA solution. The solution was mixed with 0.25 volume of 5 mM DBCO-sulfo-biotin (BP22296, BroadPHARM) in DMSO and incubated for 1 h at 25°C.

### Preparation of 3′-dialdehyde RNAs

The RNA was oxidized to 3′-dialdehyde by NaIO_4_. 1 μM RNA was incubated with 10 mM NaIO_4_ on ice in the dark for 1 h in the buffer containing 50 mM sodium acetate (pH = 5.2), 150 mM NaCl, 10 mM MgCl_2_ and 0.1 mM EDTA.

### *In vitro* selection based on click reaction

*In vitro* selection based on click reaction was carried out as follows: 33.3 μM (for the first round) or 3.33 μM RNA library was subject to self-aminoacylation and followed by a copper-free click, using the method described in the previous sections. The RNA was precipitated by ethanol and resuspended in 100 μl Tx binding buffer (50 mM HEPES–KOH (pH = 7.5), 500 mM KCl, 5 mM EDTA, 0.05% tween).

Then, the RNA solution was mixed with 20 μl Dynabeads MyOne Streptavidin C1 (65001, Life Technologies) prewashed by Tx binding buffer three times before use. The mixture was incubated at 4°C for 30 min with agitation. After incubation, the supernatant was discarded, and beads were washed with 200 μl of Tx binding buffer, 6 M urea with 0.05% tween, and then 0.05% tween. Washed beads were resuspended in 50 μl of Tx elution buffer (50 mM HEPES-KOH (pH = 7.5), 300 mM NaCl, 5 mM EDTA, 1 mM biotin, 0.05% tween) and heated at 95°C for 10 min. The supernatant was collected and precipitated with ethanol.

The RNA pellet was resuspended in 5 μl water and reversed transcribed with oligo 4 by Superscript IV (18090050, Invitrogen), following the manufacturer's protocol. To quantify the amount of recovered RNA, qPCR was performed. 1% of the resulting reversed-transcription solution was mixed with qPCR mix (1 μM oligo 4, 1 μM oligo 5, 0.2 mM dNTP, 1:20 000 dilution of SYBR Green I (50512, Lonza), Taq polymerase Hot Start Version (R007A, TaKaRa) and 1× PCR buffer). qPCR was performed on a LightCycler Nano (Roche Diagnostics) by 25 cycles of 95°C 30 s (5°C/s), 55°C 30 s (4°C/s) and 72°C 60 s (0.5°C/s) to measure *C_q_* values.

The remaining reverse-transcription solution was mixed with 200 μl qPCR mix without SYBR Green I, and the cDNA was PCR amplified for *C_q_*-1 cycles of 95°C 30 s, 55°C 30 s, 72°C 60 s. DNA was extracted with a phenol-chloroform mixture and then precipitated with ethanol for the next rounds of selection.

### *In vitro* selection using b-tREX

*In vitro* selection using b-tREX was carried out as follows: 3.33 μM RNA library was subject to aminoacylation, followed by NaIO_4_ oxidation as described in the previous sections. The resulting RNA library was then precipitated by ethanol and resuspended in the buffer containing 0.05 M bicine (pH = 9.0), followed by incubation at 42°C for 1 h to deacylate the attached amino acid. Deacylation was quenched by adding 1/10 volume of 3 M NaOAc (pH = 5.2), and the resulting RNA library was precipitated with ethanol and resuspended with water.

Extension reaction was carried out by Klenow fragment, exo- (M0212, NEW ENGLAND Biolabs), in the presence of 1× NEBuffer 2, 2 μM RNA library, 2 μM oligo 6 and 20 μM biotin-11-dUTP (R0081, Thermo Scientific). After the extension, the solution was subjected to DNase (M6101, Promega) treatment to remove oligo 6. The resulting RNA was precipitated with ethanol and resuspended in 100 μl Tx binding buffer. The solution was subjected to Dynabeads MyOne Streptavidin C1 binding, elution, reversed transcription, and PCR amplification as described in the previous click reaction-based *in vitro* selection.

### Sequencing

The selected library was sequenced using Miseq Reagent kit v3 (150-cycle, MS-102-3001, Illumina). The randomized anti-terminator region of the cDNA generated from *in vitro* selection was first enriched by oligo 7 and 8, then subjected to second-tailed-PCR using oligo 9 and 10, following the adapter design described in the Miseq library prep kit.

### Analysis of aminoacylation by SAv-EMSA

RNA aminoacylated with Phe^AZ^-CME and clicked with DBCO-sulfo-biotin was precipitated by ethanol and dissolved in SAv loading buffer (0.5 mM sodium acetate (pH = 5.2), 4 M urea, 1 mM Na_2_EDTA, 1 mM Tris, 1.67 mg/ml streptavidin (Z7041, Promega)). The resulting solution was loaded on denaturing PAGE (8 M urea, 1× TBE). After electrophoresis, gels were stained with ethidium bromide and imaged with Typhoon FLA 7000 fluorescence image analyzer (GE Healthcare). Aminoacylation efficiency was determined from the band intensity of SAv-binding RNAs (S) and free RNA (F) and calculated as S/(S + F).

### Analysis of aminoacylation by EMSA using acid PAGE

Aminoacyl-tRNA was prepared, and ethanol precipitated according to the protocol shown in previous sections. The resulting pellet was dissolved in the acid PAGE loading buffer (150 mM sodium acetate (pH = 5.2), 10 mM EDTA (pH = 8.0), and 93% (vol/vol) formamide), and then loaded onto 12% acrylamide acid PAGE (8 M urea, 50 mM sodium acetate (pH = 5.2)). Electrophoresis was performed at 180 V (approximately 12 V∙cm^−1^) for 12 h at 4°C. After electrophoresis, gels were stained with ethidium bromide and imaged with Typhoon FLA 7000 fluorescence image analyzer (GE Healthcare). Aminoacylation efficiency was determined from the band intensity of aminoacyl-tRNA (A) and free tRNA (F) and calculated as A/(A + F).

### Primer extension

0.75 μM oligo 23 was labeled with ^32^P by T4 polynucleotide Kinase (2021A, TaKaRa) in the presence of adenosine 5′-triphosphate, [γ-^32^P] (6000 Ci/mmol, 10 mCi/ml, NEG502Z, Perkin Elmer), following the manufacturer's protocol. 5′-^32^P labeled primer was purified by denaturing PAGE containing 8 M urea and 1× TBE. Purified primer was stocked in 1 mM HEPES-KOH (pH = 8.0).

inT1-at-s was first aminoacylated in the presence or absence of Phe^AZ^-CME, precipitated by ethanol, and resuspended in 0.5× TE buffer (5 mM Tris, 0.5 mM EDTA, pH = 7.4). Both inT1-at-s samples were reverse transcribed by Superscript IV (18090050, Invitrogen) following the protocol described in (53), while the concentration of each dNTP was adjusted to 10 μM to induce reverse transcription stop.

For the ladders to identify the reverse transcription stop site, non-aminoacylated inT1-at-s was reverse transcribed by Superscript III (18080044, Invitrogen) following the protocol described in (53). In each entry, 1 mM of ddATP, ddTTP, ddCTP or ddGTP was added to the reaction to stop reverse transcription randomly.

Reactions were quenched and loaded onto denaturing PAGE (7 M urea, 1 × TBE) and run at 50 W for 4 h. After electrophoresis, the gel was exposed to an IP cassette (Fujifilm) and imaged by Typhoon FLA 7000 fluorescence image analyzer (GE Healthcare).

### Mass spectrometry for RNA modification analysis

Mass spectrometric analysis was conducted based on a previous study (54). For RNA fragment analysis, 3 pmol of inT1-at-s was digested in a 10 μl mixture containing 20 units of RNase T_1_ (Thermo Fisher Scientific) and 25 mM NH_4_OAc (pH 5.3) at 37°C for 1 h. The reaction mixture was mixed with 10 μl of 100 mM triethylamine (TEA) acetate (pH 7.0) and analyzed by an LC/MS system (DiNa splitless nano HPLC system (Techno Alpha Co., Ltd) and LTQ Orbitrap XL (Thermo Fisher Scientific)). The RNase T_1_-digests were loaded on a trap column (L-column2 ODS, 5 μm particle, 0.3 × 5 mm; Chemical Evaluation and Research Institute) and separated on a HiQ sil C18W-3 column (C18, 3 μm particle, 0.1 × 100 mm; Techno Alpha Co., Ltd) in 0.4 M 1,1,1,3,3,3-hexafluoro-2-propanol (pH 7.0, adjusted with TEA) with a linear gradient of 2.5–50% methanol over 35 min at a flow rate of 300 nl/min. Negative ion scanning ranged from 600 to 2000 *m/z*.

For nucleoside analysis, 15 pmol of inT1-at-s was digested at 37°C for 1 h in a 10 μl mixture containing 50 mM trimethylamine-acetate (pH 7.0), 0.5 U of nuclease P1 (Fujifilm Wako Pure Chemical), and 0.5 U of alkaline phosphatase (*E. coli* C75; Takara Bio). Acetonitrile was added to the reaction up to 90% final concentration, then analyzed by an LC/MS system (UltiMate 3000 LC (Thermo Fisher Scientific) and Q Exactive (Thermo Fisher Scientific)) (55). The digests were separated on a ZIC-cHILIC column (3 μm particle, 2.1 × 150 mm; Merck Millipore) with a guard column (3 μm particle, 2.1 × 20 mm, Merck Millipore). The solvent system comprised 5 mM NH_4_OAc (pH 5.3) (solvent A) and acetonitrile (solvent B) with a multi-step gradient (90–40% B from 0 to 30 min, 40% B for 10 min, and then initialized to 90% B) at a flow rate of 100 μl/min. Positive ion scanning ranged from 110 to 700 *m/z*. Data were analyzed using the Xcalibur Qual Browser (Thermo Fisher Scientific).

### Measurement of initial aminoacylation rate

tRNA used to measure the initial *trans*-aminoacylation rate was body-radiolabeled by *in vitro* transcription in the presence of [α-^32^P]-UTP (800 Ci/mmol, 10 mCi/ml, NEG507X, Perkin Elmer). The conditions for *trans*-aminoacylation were the same as previously described. At each time point, the reaction solution was aliquoted and mixed with ethanol to stop the reaction, then precipitated. The resulting pellet was then suspended in the biotin-labeling mixture (0.66 μM sodium acetate (pH = 5.2), 0.06 M HEPES-KOH (pH = 8.0), 6.67 μg/μl sulfo-biotin-NHS (21217, Thermo Scientific), 13.3% DMSO) and kept on ice for 1.5 h to label Phe^AZ^-tRNA with biotin. After the reaction, the solution was stopped by ethanol precipitation. The resulting pellet was resuspended in the SAv loading buffer and loaded on denaturing PAGE (8 M urea, 1× TBE). After electrophoresis, gels were exposed to an IP cassette (Fujifilm) and imaged by Typhoon FLA 7000 fluorescence image analyzer (GE Healthcare). The initial rate was determined *n* = 3 by the slope of the linear region in a plot of reaction time versus product concentration with four time points. The initial rate at each substrate concentration was fitted to the Michaelis–Menten nonlinear curve with Prism (GraphPad Software) to determine the kinetic parameters.

### *In vitro* translation

The FIT system (51), which contains all components necessary for *in vitro* translation, was used. The components of the FIT system were as below: 50 mM HEPES–KOH (pH 7.6), 19.5 mM magnesium acetate, 100 mM potassium acetate, 2 mM spermidine, 20 mM creatine phosphate, 2 mM DTT, 2 mM ATP, 2 mM GTP, 1 mM CTP, 1 mM UTP, 0.1 mM 10-formyl-5,6,7,8-tetrahydrofolic acid, 0.5 mM Tyrosine and Lysine, 50 μM Aspartic acid and 1.5 mg/ml *E. coli* total tRNA along with 0.73 μM AlaRS, 0.03 μM ArgRS, 0.38 μM AsnRS, 0.13 μM AspRS, 0.02 μM CysRS, 0.06 μM GlnRS, 0.23 μM GluRS, 0.09 μM GlyRS, 0.02 μM HisRS, 0.4 μM IleRS, 0.04 μM LeuRS, 0.11 μM LysRS, 0.03 μM MetRS, 0.68 μM PheRS, 0.16 μM ProRS, 0.04 μM SerRS, 0.09 μM ThrRS, 0.03 μM TrpRS, 0.02 μM TyrRS, 0.02 μM ValRS, 0.6 μM MTF, 2.7 μM IF1, 3 μM IF2, 1.5 μM IF3, 0.26 μM EF-G, 10 μM EF-Tu, 10 μM EF-Ts, 0.25 μM RF2, 0.17 μM RF3, 0.5 μM RRF, 0.1 μM T7 RNA polymerase, 4 μg/ml creatine kinase, 3 μg/ml myokinase, 0.1 μM pyrophosphatase, 0.1 μM nucleotide-diphosphatase kinase, 1.2 μM ribosome. Additional components’ final concentrations were shown as follows: 8 mM Phe^AZ^-CME (in DMSO, 10% (vol/vol)), 50 μM elT32 or 50 μM elT49, 50 μM tRNA_GGC_, 200 nM DNA template.

The translation was incubated at 37°C for 1 h. eFx-catalyzed Phe^AZ^-tRNA was prepared as described in (35) and was added to the translation mixture at 50 μM final concentration to be the positive control for peptide translation.

### Tricine SDS-PAGE analysis of translation products

The translation mixture was performed in the presence of 50 μM l-[^14^C(U)]-Aspartic acid (>200 mCi/mmol, 0.1 mCi/ml, NEC268E, Perkin Elmer). After translation, the solution was mixed with 2x tricine SDS-PAGE loading buffer (0.9 M Tris–HCl (pH = 8.45), 8% SDS, 30% glycerol, and 0.001% xylene cyanol) and subjected to 15% tricine SDS-PAGE gels at 150 V for 40 min. The resulting gel was exposed to an IP cassette (Fujifilm) and imaged by Typhoon FLA 7000 fluorescence image analyzer (GE Healthcare).

### MALDI-TOF MS analysis

After translation, the mixture was mixed with 2× TBS buffer (100 mM Tris–HCl (pH = 7.6), 300 mM NaCl). The solution was then subjected to 10 μl prewashed Anti-FLAG M2 affinity gel (A2220, Sigma) and incubated with agitation at room temperature for 1 h. After incubation, the gel beads were washed with 50 μl of 1× TBS (50 mM Tris–HCl (pH = 7.6), 150 mM NaCl), and the peptide was eluted out with 10 μl 0.2% trifluoroacetic acid. The peptide solution was desalted with a C18 tip (Nikkyo Technos) and analyzed by ultrafleXtreme MALDI-TOF MS (Bruker Daltonics) in reflector-positive mode. External calibration was carried out by Peptide Calibration Standard II (Bruker Daltonics).

## Results and discussion

### *In vitro* selection of aminoacylation ribozymes by a click reaction-based method

**Figure 1.**
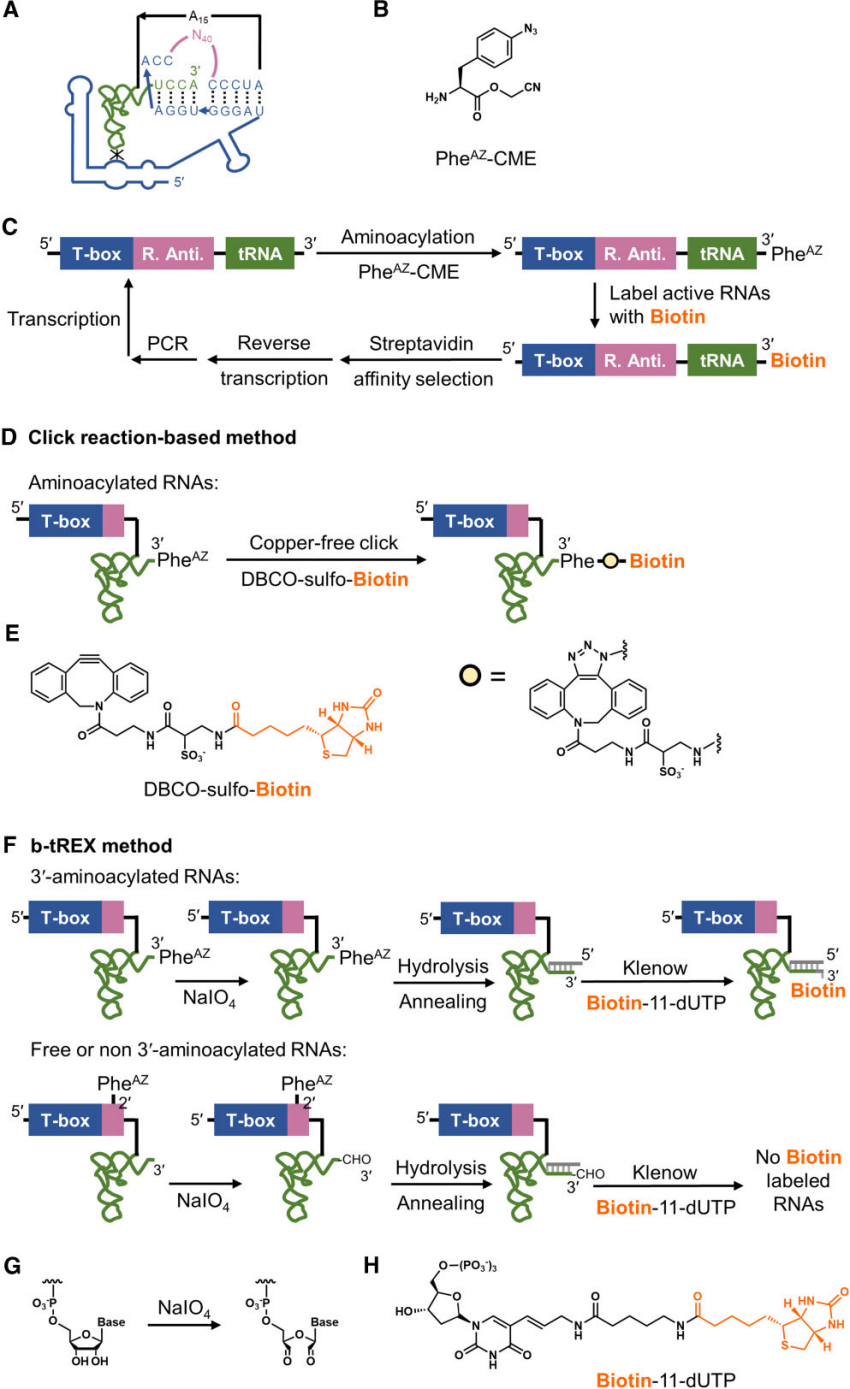
Strategies to obtain tRNA 3′-aminoacylation ribozymes. (**A**) Schematic illustration of the library containing a 40-nt randomized anti-terminator region derived from *B. subtilis glyQS* T-box riboswitch with its unbound cognate tRNA^Gly^. Blue indicates the T-box riboswitch scaffold, and green represents its cognate tRNA. The randomized anti-terminator region is colored in pink, and the A15 linker is in black. (**B**) Chemical structure of para-azido-l-phenylalanine cyanomethyl ester (Phe^AZ^-CME), the amino acid substrate used in this study. (**C**) General scheme of *in vitro* selection based on the specific interaction between biotin and SAv. Biotin is colored orange. R. Anti., randomized anti-terminator region. (**D**) Click reaction-based method using SPAAC between azido group and DBCO-sulfo-biotin to label self-aminoacylated RNA population with biotin for SAv affinity selection. (**E**) The chemical structure of DBCO-sulfo-biotin used in this study. The click reaction product is shown and abbreviated as a yellow dot. (**F**) b-tREX method based on NaIO_4_ oxidation of RNA 3′-vicinal diols. While 3′-aminoacylated RNAs survive the oxidation and later get a biotin label during the Klenow fragment, exo- extension in the presence of biotin-11-dUTP, free or non-3′-aminoacylated RNAs are oxidized into 3′-dialdehydes and no oxidized RNAs can get a biotin label. (**G**) Mechanism of NaIO_4_ oxidation resulting in RNA 3′-dialdehydes. (**H**) Chemical structure of biotin-11-dUTP used in this study.

To develop new tRNA-aminoacylation ribozymes, a randomized RNA library derived from *B. subtilis glyQS* T-box riboswitch (Supplementary Figure S1) was prepared as described in the previous discovery of T-boxzymes (50). P8 anti-terminator domain of *B. subtilis glyQS* T-box, whose deletion does not affect cognate tRNA binding (56), was substituted with a 40-nucleotide (nt) randomized sequence. Its cognate *B. subtilis* tRNA^Gly^_GCC_ was linked to the 3′-end of the T-box derived RNA library via a poly-adenosine (A15) linker (Figure 1A).

For the evolution of T-boxzymes adopting *N*-termini-free substrates, Phe^AZ^-CME was chosen as the amino acid substrate (Figure 1B). The CME group is a weakly activated leaving group, yet stable under buffered conditions at near neutral pH (20,57). Phenylalanine was chosen as a representative amino acid sidechain since its aromatic ring benefits from hydrophobic/π–π stacking interactions with nucleobases. Based on this property, such a phenylalanine sidechain would be suitable for ribozyme selections (10–15,17,19,22,23,25,50).

The *in vitro* selection was carried out based on the strong interaction between biotin and streptavidin (SAv) (Figure 1c). During one round of *in vitro* selection, RNA population aminoacylated with Phe^AZ^-CME would be labeled with biotin through a strain-promoted [3 + 2] azide−alkyne cycloaddition (SPAAC) (58), so-called click reaction, with dibenzocyclooctyne-sulfo-biotin (DBCO-sulfo-biotin, Figure 1D, E). Then, the clicked RNA population was selectively recovered by biotin-SAv interaction, reverse transcribed (RT), and amplified by polymerase chain reaction (PCR) for the next rounds of selection.

### Self-aminoacylation of outcome library and characterization of top inT-boxyzme

**Figure 2.**
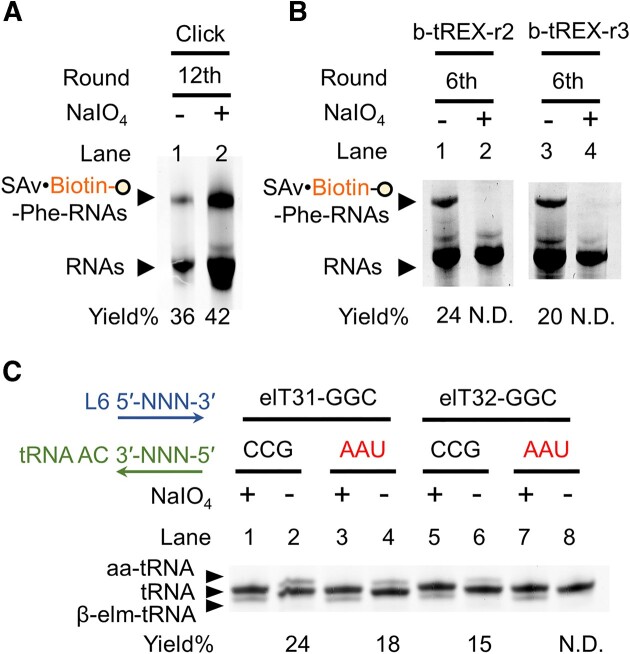
Outcomes of combined *in vitro* selection. The RNA libraries from the last round of (**A**) click reaction-based selection and (**B**) b-tREX selections were tested for self-aminoacylation activity by SAv-EMSA. (**C**) NaIO_4_ oxidation confirmed that both elT31 and elT32 catalyzed tRNA 3′-aminoacylation. Two elT-boxzymes were also tested for their tRNA anticodon selectivity. AC, anticodon. aa-tRNA, aminoacylated tRNA. β-elm-tRNA, β-eliminated tRNA. N.D., non-detected.

After 12 rounds of *in vitro* selection of self-modifying species with Phe^AZ^-CME, the rate of RNA recovery from SAv-immobilized beads increased (Supplementary Figure S2). To confirm whether the selected population was catalyzing 3′-aminoacylation, we applied sodium periodate (NaIO_4_) oxidation to the outcome RNA library before carrying out aminoacylation reaction. The RNA populations bearing 3′-vicinal diols or 3′-dialdehydes were then aminoacylated and clicked with DBCO-sulfo-biotin, and later analyzed by SAv-dependent electrophoretic mobility shift assay (SAv-EMSA). The aminoacylated populations as the shifted band did not significantly change regardless of NaIO_4_ oxidation (Figure 2A, lanes 1, 2). This indicated that most of the enriched RNA population was likely aminoacylated at a 2′-internal hydroxyl group rather than the 3′-terminal hydroxy group. Because of this catalytic property, we referred to them as inT-boxyzmes.

The RNA library from the last round of *in vitro* selection was analyzed by deep sequencing. A consensus motif, AAUGAAAUAA, was identified by aligning the top sequences with the most reads (Supplementary Figure S3). The most abundant sequence of inT-boxyzme (inT1) conjugated with tRNA, inT1-tRNA, was chosen as a representative to identify the aminoacylation site (Supplementary Figure S4a). To facilitate the identification, we truncated inT1-tRNA to find shorter catalytic derivates. Three variants of inT1-tRNA were prepared: the full-length [inT1-tRNA] (G1–tA75, ‘t’ stands for tRNA numbering), the T-box part [inT1] (G1–A194), and the anti-terminator region [inT1-at] (U138–A194). Each of their activity was analyzed by SAv-EMSA (Supplementary Figure S4b). Three variants all showed self-aminoacylation activity, which agreed with their 2′-internal aminoacylation activity.

We then applied primer extension assay to the shortest derivate, inT1-at, to locate the aminoacylation site. We added flanking sequences (53) to both termini of inT1-at to prevent information loss during RT, which resulted in ‘inT1-at-s’ (Supplementary Table S1). An extra band of RT stop was observed in the aminoacylated entry compared with the non-aminoacylated one (treated with only DMSO, Supplementary Figure S5a). Referring to the ladder, the RT stop site was read as U167, located inside the consensus motif of AAUGAAAUAA.

To confirm the Phe^AZ^ acylation site at a molecular level, we further carried out mass spectrometry analysis on inT1-at-s with reference to the non-aminoacylated one. In the sample of RNase T_1_-digested Phe^AZ^-inT1-at-s, we found an extra enriched peak whose mass corresponded to AAAUAAAAGp + 188 Da, while in the negative sample, only the peak of mass for AAAUAAAAGp was detected (Supplementary Figure S5b, upper panel). This mass difference should correspond to the RNA fragment charged with Phe^AZ^. Its collision-induced dissociation (CID) analysis showed the occurrence in the UA region of AAAUAAAAGp (Supplementary Figure S5b, lower panel). Then, nucleoside analysis of the Phe^AZ^-inT1-at-s by treating nuclease P1 and bacterial alkaline phosphatase gave an extra peak corresponding to U + 188 Da compared with the negative sample (Supplementary Figure S5c, upper panel). An unmodified uracil base was also detected by CID analysis from the U + 188 Da product (Supplementary Figure S5c, lower panel). Therefore, we concluded that the inT1 self-acylation site with Phe^AZ^-CME was the 2′-OH group in the ribose of U167 inside the consensus motif (Supplementary Figure S5d, e). The AAUGAAAUAA motif differs from previously reported internal acyl-transfer ribozymes (11,16–18), which suggests a possibility of widespread ribozyme catalysis for RNA-amino acid conjugates during the early stage of life.

### *In vitro* selection by a biotin-based tRNA extension (b-tREX) method

The disappointing outcome of the selective enrichment of the inT-boxyzmes led us to devise an alternative method that selectively enriches the desired tRNA 3′-aminoacylation ribozymes. An approach named tRNA extension (tREX) was previously reported to screen the 3′-aminoacylation status of each tRNA by DNA-templated RNA extension (59). Inspired by this approach, we elaborated an *in vitro* selection-capable strategy, named biotin-based tRNA extension (b-tREX), to pursue elT-boxyzmes (Figure 1F). In one round of b-tREX, the RNA library was subjected to aminoacylation followed by NaIO_4_ oxidation. This step selectively made non-3′-aminoacylated species incapable of the following extension reaction (Figure 1G). After oxidation, deacylation was carried out to release attached amino acids. The RNA library was then annealed to the designed DNA oligo bearing a 5′-AAA overhang. Later, the Klenow fragment, exo- extension in the presence of biotin-11-dUTP (Figure 1H) labeled non-oxidized RNAs with biotin. Therefore, RNA sequences catalyzing aminoacylation at the 3′-terminus of tRNA would be selectively labeled with biotin and then captured by SAv-immobilized magnetic beads. These recovered elT-boxyzme-tRNA conjugates were converted to DNA by RT and amplified by PCR for the next rounds of selection.

This b-tREX method was applied to the populations selected by the click reaction-based method (Supplementary Figure S6a). Two selection campaigns for the population after round 2 (b-tREX r2) and after round 3 (b-tREX r3) were carried out in parallel. After an additional six rounds of b-tREX selection, the RNA recovery rate increased and reached a plateau (Supplementary Figure S6b, c). SAv-EMSA showed that the RNA populations from both b-tREX r2 and r3 had self-aminoacylation activity, and their activity was abolished by NaIO_4_ oxidation (Figure 2B, lanes 2, 4). This result suggested that the populations of the ribozymes catalyzing the 3′-aminoacylation, namely the candidates of elT-boxzymes, were selectively enriched by the b-tREX method as expected.

### *Trans*-aminoacylation activity of elT-boxzymes

The RNA populations from the last four rounds of both b-tREX selections were analyzed by deep sequencing. The top 1000 sequences with the most reads in each RNA library were aligned. Among the alignment, we identified 36 unique elT-boxzyme candidates from distinct families. The T-box part (G1–A194) of these 36 clones was named elT01 to elT36, sorted by descending reads (Supplementary Table S1). We then tested their *trans*-aminoacylation activity onto tRNA. EMSA revealed that only three candidates, elT09, elT31 and elT32, were active in *trans*, while elT09 only showed a modest conversion (≤10%) of tRNA aminoacylation (Supplementary Figure S7).

In natural *B. subtilis glyQS* T-box, the triplet bases in loop 6 (L6, G87–G88–C89), referred to as the ‘specifier sequence’, form complementary base-pairs with tRNA anticodon (tG34–tC35–tC36), and thus contribute to *B. subtilis glyQS* T-box's specific tRNA recognition (48,49). We, therefore, investigated whether elT31 and elT32 inherited the interaction between specifier sequence and tRNA anticodon by evaluating their *trans*-aminoacylation activity towards tRNA bearing a noncognate UAA anticodon. The *trans*-aminoacylation activity of elT31 was retained regardless of cognate/noncognate tRNAs (Figure 2C, lanes 2, 4), whereas that of elT32 was abolished with the noncognate tRNA (Figure 2C, lanes 6, 8). Since elT32 exhibited the aminoacylation activity in response to the complementary interaction between specifier sequence and cognate tRNA anticodon, we focused on this particular elT-boxzyme for further studies.

### *In vitro* evolution of elT32

The elT32 did act as a tRNA-anticodon sensing *trans*-aminoacylation ribozyme, yet it formed Phe^AZ^-tRNA in an unsatisfying yield (15% conversion, Figure 2C, lane 6). Our goal for this study was to develop an elT-boxzyme that functions *in situ* in our FIT system to incorporate Phe^AZ^ into the nascent peptide chain. However, we were concerned that such a modest yield of Phe^AZ^-tRNA might suffer from the competition with the residual natural aminoacyl-tRNA cognate or near-cognate to the codon reprogrammed to Phe^AZ^, resulting in misincorporations. Based on our past experiences, such unwanted ‘background misincorporations’ largely depend on the codon used (60). We thus explored the specifier sequence of elT32 and the anticodon of tRNA to determine which codon pair could be used to maintain the tRNA-sensing aminoacylation activity of elT32 and, at the same time, to determine if the reprogrammed codon has a low background misincorporation.

We first flipped the elT32 specifier sequence with its cognate tRNA anticodon to determine what pairs were tolerable by elT32 aminoacylation. Eight kinds of GC-containing specifier sequences and anticodons were prepared and tested for their *trans-*aminoacylation (Supplementary Figure S8a). The elT32 mutant bearing GCC specifier sequence exhibited the same level of *trans-*aminoacylation activity to its cognate tRNA_GGC_ (15%, lane 5) as the parental elT32 bearing GGC to tRNA_GCC_ (15%, lane 7, highlighted in red), while elT32 bearing CGC showed a reduced conversion rate (9%, lane 3). Other combinations resulted in a complete loss of the activity.

We then investigated which of these three elongation codons had lower ‘background misincorporations’ during translation, *i.e*. lower competition with the natural cognate/ near-cognate aminoacyl-tRNAs. DNA templates bearing either of the three elongation codons [CGC (Arg), GCC (Ala), and GGC (Gly)] were prepared (Supplementary Figure S8b). We could expect that no full-length peptide (P1-3) was translated from the mRNAs (mR1-3) in an ‘exhausted’ FIT system having only four amino acids (Met, Lys, Asp, and Tyr), *i.e*. depletion of Arg, Ala, and Gly. In the case of CGC and GCC codons in mR1 and mR2, we observed a small peak of the full-length peptide containing Arg and Ala, respectively, indicating that trace amounts of Arg and Ala were present in the FIT system (Supplementary Figure S8b). Besides the full-length peptide, no other peak was detected. This suggests that if Phe^AZ^ could be effectively charged onto the tRNA_GCG_ or tRNA_GGC_ by an elT-boxzyme *in situ* of the FIT system, it is possible to suppress the background competition and incorporate Phe^AZ^ at the designated codon. In contrast, in the case of the GGC codon in mR3, we observed not only the full-length peptide containing Arg but also Asp, suggesting that this codon might introduce its misincorporation (Supplementary Figure S8b). Based on the above results, we decided to use GCC codon for Phe^AZ^ incorporation, and thereby elT32 bearing the GCC specifier sequence (elT32_GCC_) paired with tRNA_GGC_ for future study.

**Figure 3.**
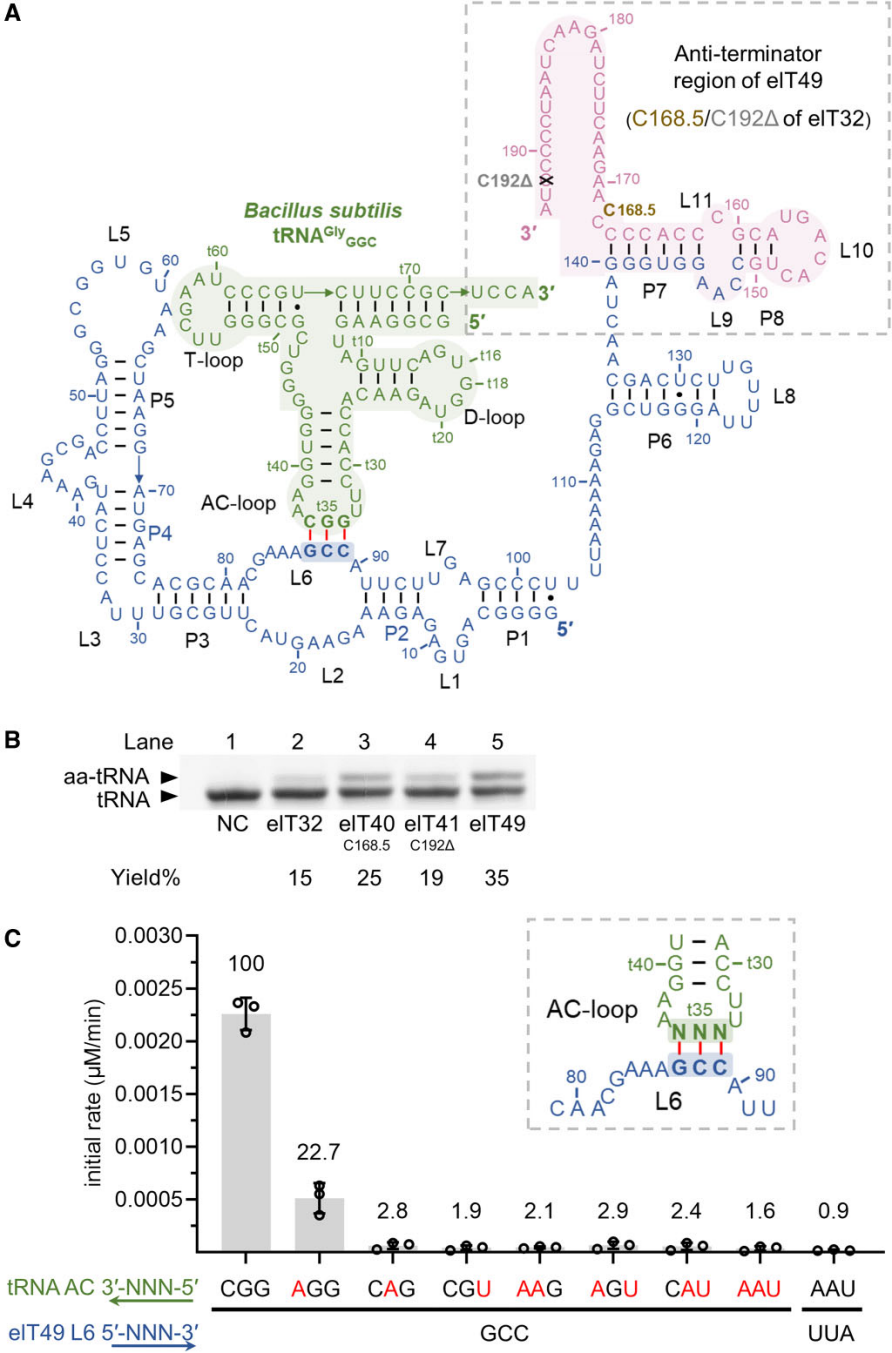
Characterization of elT49, with enhanced aminoacylation activity, evolved from elT32. (**A**) A predicted secondary structure of elT49. Coloring follows Figure 1. The numbering follows that of elT32. The ‘t’ placed before numbers stands for tRNA numbering. The brown letter denotes insertion, while the grey denotes deletion. (**B**) *Trans*-aminoacylation activity of elT32-mutants and elT32 in the presence of 5 mM Phe^AZ^-CME. Negative control (NC) was performed with elT32 without Phe^AZ^-CME. aa-tRNA, aminoacylated tRNA. (**C**) Apparent initial rates of elT49 *trans*-aminoacylation against tRNAs bearing various anticodons. Red letters stand for mismatched base pairs. Each initial rate was measured under 5 mM Phe^AZ^-CME, determined by the mean of *n* = 3, and normalized to the correct pair's activity, set as 100. Each circle represents a measurement. Error bars show standard deviation.

We then designed focus libraries for the *in vitro* elT32_GCC_ evolution based on its anti-terminator region's predicted secondary structure (61) (U138–A194, Supplementary Figure S9a). Two predicted loops were randomized with varied sizes, resulting in a combination of nine different libraries (Supplementary Figure S9b). The last 27-nt in the anti-terminator region predicted to be flexible was also fully randomized (Supplementary Figure S9c). The click reaction-based selection was carried out for the evolution. To mimic the conditions of the FIT system, the potassium concentration for self-aminoacylation was switched from 500 mM KCl to 100 mM KOAc. After four selection rounds, the RNA recovery nearly plateaued (Supplementary Figure S10). Thus, RNA populations from each round were forwarded to the deep sequencing analysis for their sequence alignment. Although the most abundant RNA population was the same as the parental elT32, we picked out 12 variants having a single mutation. These clones were named elT37 to elT48, sorted by descending reads (Supplementary Table S1). These variants were independently tested for their *trans*-aminoacylation activity (Supplementary Figure S11). Although the last 27-nt (C168–A194) in the anti-terminator region of elT32 was predicted to be structurally undefined, some of their base mutations could abolish the activity, suggesting that this region may play a role in stabilizing the core structure of elT32. Among them, elT40_C168.5 and elT41_ C192Δ showed 1.7-fold and 1.3-fold enhancements of their parental elT32 *trans*-aminoacylation activity, respectively. We then combined these two mutations (C168.5/ C192Δ) into one clone, referred to as elT49 (Figure 3A). To our fortune, elT49 showed a 2.3-fold enhancement of the activity (Figure 3B, lanes 2, 5).

### Kinetics and substrate recognition profile of elT49

To better understand elT49’s catalytic properties, we primarily focused on its kinetic parameters, *K*_M_ and *k*_cat_. The initial *trans-*aminoacylation rate of elT49 under 0.5–10 mM of Phe^AZ^-CME was measured and fitted to the Michaelis-Menten non-linear curve, where its kinetic parameters were determined to be *K*_M_= 3.6 mM, *k*_cat_= 2.0 × 10^−3^ min^−1^ (Supplementary Figure S12). The *K*_M_ value of elT49 for Phe^AZ^-CME was comparable to that of Tx2.1 for BioPhe-CME (3.0 mM), yet *k*_cat_ was 30-fold slower than Tx2.1 (5.8 × 10^−2^ min^−1^) (50).

We also investigated the anticodon specificity by disrupting the base pair between the specifier sequence and tRNA anticodon. We prepared tRNAs having single (GGA, GAC, UGC), double (GAA, UGA, UAC) and triple (UAA) mutations in the anticodon and measured their initial rates of aminoacylation (Figure 3C). Disruption of the base pair between G87–tC36 by a single mispairing mutation decreased the initial rate by 5-fold, while that in either C88–tG35 or C89–tG34 vastly reduced the initial rate to nearly negligible levels (∼2% of the correct pair's activity). In comparison, the *trans*-aminoacylation activity of Tx2.1 was reduced only 1.5-fold upon the same single mispairing. Thus, elT49 was far less permissible of mismatch anticodons, i.e. more sensitive to the specifier sequence-anticodon interaction. We also prepared a new compensation mutation pair of tRNA_UAA_ and elT49_UUA_, but it showed no sign of activity recovery (Figure 3C, UUA-UAA). This was again in sharp contrast to Tx2.1, which keeps about 20% of aminoacylation activity with the same compensation mutants (50). This suggests that elT49 has evolved to work with only the GCC–GGC pair. This strict tRNA anticodon recognition occurring to elT49 could be reminiscent of the function of naturally occurring ARSs, expectedly granting the ability to perform *in situ* specific aminoacylation onto the elongator tRNA_GGC_ in a complex translation mixture.

To understand the scope and limitation of elT49’s amino acid substrate specificity, we also tested various aminoacyl-CME donors available in our laboratory (Supplementary Figure S13a). In addition to Phe^AZ^-CME, elT49 was able to charge its cognate tRNA_GGC_ with Phe-CME, Tyr-CME and Trp-CME in a similar yield. On the other hand, neither d-Phe-CME, Gly-CME, nor Ala-CME was the substrate for elT49. Moreover, neither hydroxy acids nor *N*-substituted Phe-CME (*N*-acetyl, *N*-biotinyl, *N*-methyl) was accepted by elT49. An alternative benzyl ester group, such as 3,5-dinitrobenzyl ester, which is the substrate for dinitro-flexizyme (dFx) (26), could not be used as an activation group (Supplementary Figure S13b). These outcomes illustrate that elT49 specifically recognizes l-amino acids having free *N*-amino and aromatic sidechains with the CME activation group (Supplementary Figure S13c). As previous Tx2.1 strictly recognizes the *N*-biotinyl and aromatic sidechains with the CME group in the aminoacyl donor (50), elT49 is a T-boxzyme distinctive from Tx2.1 even though derived from the same library.

### Integration of elT-boxzyme with *in vitro* translation

**Figure 4.**
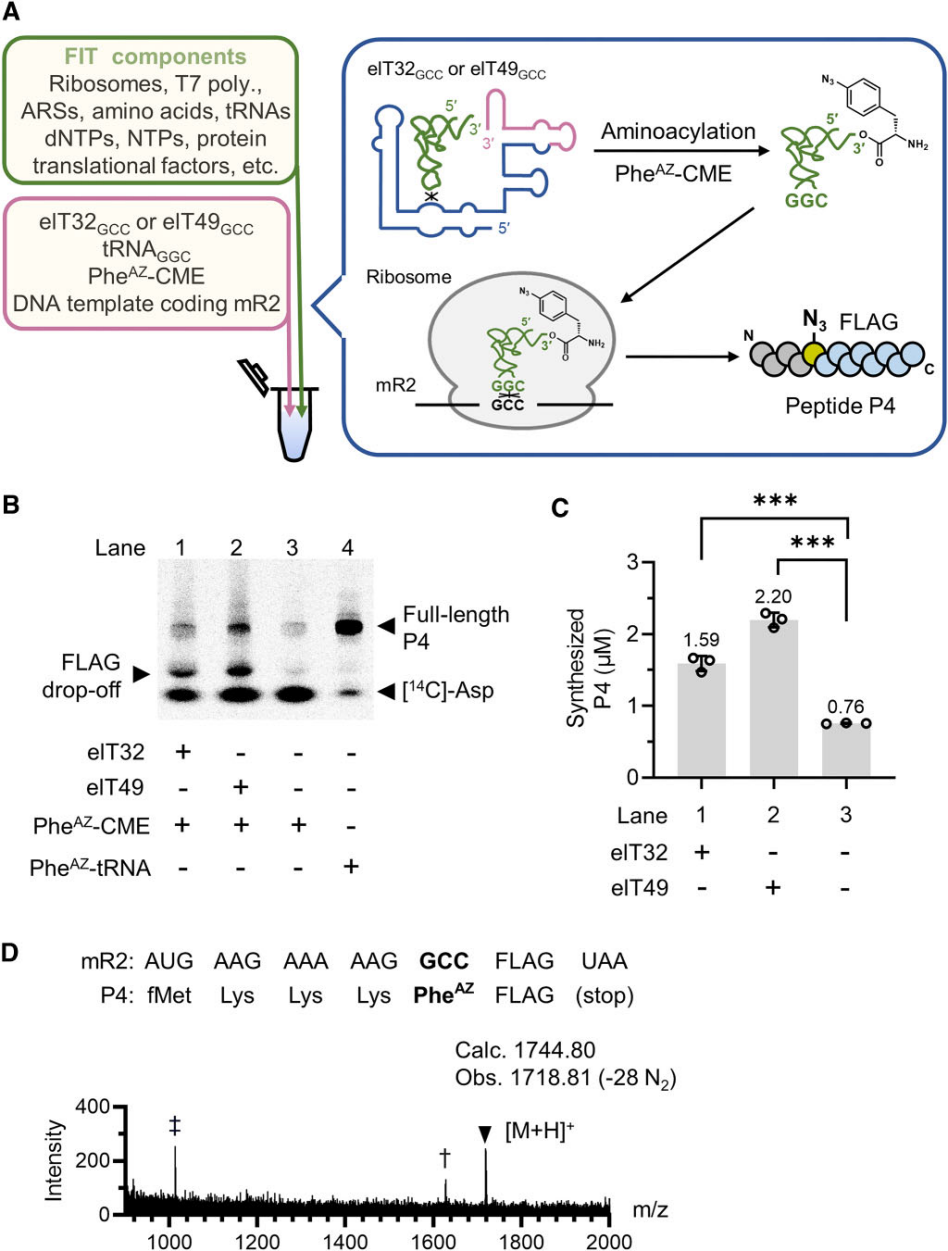
Integrating elT32 and elT49 with a custom-made translation system. (**A**) One-pot *in vitro* translation carried out in this study. The FIT system contained elT-boxzyme (elT32_GCC_ or elT49_GCC_), tRNA_GGC_, Phe^AZ^-CME, and a DNA template coding mR2 for the model Phe^AZ^-containing peptide, P4. (**B**) Representative [^14^C]-Asp autoradiography of the translation products separated by tricine SDS-PAGE. Lanes 1 and 2 were one-pot *in vitro* translation in the presence of either elT32 or elT49, while lane 3 was in the absence of elT-boxzyme. Lane 4 is the positive control where full-length P4 was produced in the presence of Phe^AZ^-tRNA_GGC_ prepared by flexizyme (eFx). (**C**) Quantification of P4 synthesized in lanes 1–3 in (B). The values were determined by the mean of *n* = 3. Each circle represents a measurement. Error bars show standard deviation. (**D**) MALDI-TOF MS of the translational product from lane 2. Arrowhead indicates the product P4, where its azido group was reduced into nitrene by laser and resulted in the observation of the peak [M + 1–28]^+^ (61). †, the peak corresponding to the peptide in which alanine remaining in the FIT mixture incorporated into the GCC codon. ‡, the peak corresponding to the peptide dropped off at the GCC codon, whose sequence was FLAG (DYKDDDDK). Calc., calculated; Obs., observed.

As we have achieved novel elT-boxzymes with the selective recognition of the cognate tRNA and Phe^AZ^-CME, we pursued our goal for one-pot *in vitro* translation of Phe^AZ^-containing peptide by employing elT-boxzymes *in situ* aminoacylation. The FIT system (51) was customized to include elT-boxzyme (elT32_GCC_ or elT49_GCC_), tRNA_GGC_, Phe^AZ^-CME, and an mRNA (mR2) *in situ* transcribed from the corresponding DNA template (Figure 4A). mR2 bear a GCC elongation codon for Phe^AZ^ incorporation, where the elT-boxzyme would aminoacylate tRNA_GGC_*in situ* of the FIT system, resulting in a 13-mer model peptide P4 consisting of Met, Lys, Asp, Tyr, Phe^AZ^ at the designated positions (Figure 4A).

To quantify the amount of synthesized P4, we included [^14^C]-Asp into the FIT system, enabling the detection of [^14^C]-Asp autoradiography on tricine SDS-PAGE (Figure 4B). One-pot *in vitro* translation was performed in the presence of either elT32_GCC_ or elT49_GCC_ (Figure 4B, lanes 1, 2). As a positive control for the band assignment, we ran the translation of P4 in the presence of Phe^AZ^-tRNA_GGC_ prepared by flexizyme (eFx) (Figure 4B, lane 4) (26). As a negative control to detect the background level, the translation in the absence of elT-boxzymes was conducted (Figure 4B, lane 3). In the negative control, a faint band of P4 was detected (∼0.76 μM), possibly caused by the non-enzymatic formation of a trace amount of Phe^AZ^-tRNA_GGC_ (Figure 4C, lane 3). P4 was expressed in the presence of elT32_GCC_ or elT49_GCC_ by 2-fold (∼1.6 μM) or 3-fold (∼2.2 μM) over the background (Figure 4C, lanes 1, 2), respectively. MALDI-TOF MS analysis of the peptide product of lane 2 confirmed that Phe^AZ^ was successfully incorporated at the assigned GCC codon (Figure 4D). These results demonstrated that the elT-boxzymes catalyze tRNA *trans-*aminoacylation *in situ*, thus promoting target peptide production during the one-pot *in vitro* translational event.

Therefore, we filled in the missing piece of *in situ* ribozyme aminoacylation for nascent peptide chain elongation in translation. However, the translation yield achieved even by elT49 was only partially satisfying compared with Tx2.1 (specific to BioPhe-CME), which previously resulted in a 6-fold enhancement compared with background peptide synthesis (50). This should be blamed on the slow *k*_cat_ of elT49, resulting in an insufficient concentration of Phe^AZ^-tRNA generated by elT49 in the FIT system. To advance the translation efficiency by elT-boxzymes *in situ* aminoacylation, the kinetic evolution of elT49 to promote its *k*_cat_ is needed.

In conclusion, our discovery of *trans*-acting elT-boxzymes and a Tx2.1 T-boxzyme, both of which showed their tRNA aminoacylation activity in response to cognate anticodon interactions, has given strong support for the hypothesis of the existence of tRNA-aminoacylation ribozymes in primitive RNA-based translation system. After the maturation of the translation system, protein ARSs could have evolved and taken over the function of such T-boxzymes in the early stage of life.

## Supplementary Material

gkae151_Supplemental_File

## Data Availability

The data underlying this article are available in the article and in its online supplementary material. Nextgeneration sequencing data areavailable via NCBI Bioproject via accession ID PRJNA1045519. Further data are available at NCBI Genbank via the following accession IDs: PP337235–PP337284.
