# Supplementary material for: An anticodon-sensing T-boxzyme generates the elongator nonproteinogenic aminoacyl-tRNA in situ of a custom-made translation system for incorporation
Source: Nucleic Acids Res. 2024 Mar 13;52(7):3938–49. doi: 10.1093/nar/gkae151 (PMC11039980; doi:10.1093/nar/gkae151)
Supplement: gkae151_Supplemental_File [file gkae151_supplemental_file.pdf]

Supplementary information

An anticodon-sensing T-boxzyme generates the elongator nonproteinogenic aminoacyl-tRNA *in situ* of a custom-made translation system for incorporation

Wei Lu[1], Naohiro Terasaka[1,2], Yuriko Sakaguchi[3], Takeo Suzuki[3,4], Tsutomu Suzuki[3], and Hiroaki Suga[1]†

[1]Department of Chemistry, Graduate School of Science, The University of Tokyo, 7-3-1 Hongo, Bunkyo-ku, Tokyo 113-0033, Japan

[2]Earth-Life Science Institute, Tokyo Institute of Technology, 2-12-1 Ookayama, Meguro-ku, Tokyo 152-8550, Japan

[3]Department of Chemistry and Biotechnology, Graduate School of Engineering, The University of Tokyo, 7-3-1 Hongo, Bunkyo-ku, Tokyo 113-0033, Japan

[4] Department of Medical Biochemistry, Graduate School of Medicine, University of the Ryukyus, 207 Uehara, Nishihara, Okinawa 903-0125, Japan

†Corresponding author, Contact: hsuga@chem.s.u-tokyo.ac.jp

Supplementary Table 1 | Transcripts

Name	Transcripts (5'→3')
Library01	GGGGCAGUGAGAGAAAGAAGUACUUGCGUUUACCUCAUGAAAGCGACCUUAGGGCG<br>GUGUAAGCUAAGGAUGAGCACGCAACGAAAGGCAUUCUUGAGCCCUUUUAAAAAAG<br>AGGCUGGGAUUUUGUUCUCAGCAACUAGGGUGGAACCNNNNNNNNNNNNNNNNNNN<br>NNNNNNNNNNNNNNNNNNNNNCCCUAAAAAAAAAAAAAAAAGCGGAAGUAGUUCAG<br>UGGUAGAACACCACCUUGCCAAGGUGGGGGUCGCGGGUUCGAAUCCCGUCUUCCGC<br>UCCA
eFx	GGAUCGAAAGAUUUCCGCGGCCCCGAAAGGGGAUUAGCGUUAGGU
inT1-tRNA	GGGGCAGUGAGAGAAAGAAGUACUUGCGUUUACCUCAUGAAAGCGACCUUAGGGCG<br>GUGUAAGCUAAGGAUGAGCACGCAACGAAAGGCAUUCUUGAGCCCUUUUAAAAAAG<br>AGGCUGGGAUUUUGUUCUCAGCAACUAGGGUGGAACCUGAAUCUGUUAAUGAAAUA<br>AAAGCAUGAUCGCACGGACAUCCCUAAAAAAAAAAAAAAAAGCGGAAGUAGUUCAGU<br>GGUAGAACACCACCUUGCCAAGGUGGGGGUCGCGGGUUCGAAUCCCGUCUUCCGCU<br>CCA
inT1	GGGGCAGUGAGAGAAAGAAGUACUUGCGUUUACCUCAUGAAAGCGACCUUAGGGCG<br>GUGUAAGCUAAGGAUGAGCACGCAACGAAAGGCAUUCUUGAGCCCUUUUAAAAAAG<br>AGGCUGGGAUUUUGUUCUCAGCAACUAGGGUGGAACCUGAAUCUGUUAAUGAAAUA<br>AAAGCAUGAUCGCACGGACAUCCCUA
inT1-at	GGGUAGGGUGGAACCUGAAUCUGUUAAUGAAAUAAAAGCAUGAUCGCACGGACAUC<br>CCUA
inT1-at-s	GGCCUUCGGGCCAAUAGGGUGGAACCUGAAUCUGUUAAUGAAAUAAAAGCAUGAUC<br>GCACGGACAUCCCUAUCGAUCCGGUUCGCCGGAUCCAAAUCGGGCUUCGGUCCGGU<br>UC
eIT01	GGGCAGUGAGAGAAAGAAGUACUUGCGUUUACCUCAUGAAAGCGACCUUAGGGCGG<br>UGUAAGCUAAGGAUGAGCACGCAACGAAAGGCAUUCUUGAGCCCUUUUAAAAAAGA<br>GGCUGGGAUUUUGUUCUCAGCAACUAGGGUGGAACCCCUCUACGUCUGAGUAGGAC<br>GUGCAGGCUUCCUUUAGAUACCCUA
eIT02	GGGGCAGUGAGAGAAAGAAGUACUUGCGUUUACCUCAUGAAAGCGACCUUAGGGCG<br>GUGUAAGCUAAGGAUGAGCACGCAACGAAAGGCAUUCUUGAGCCCUUUUAAAAAAG<br>AGGCUGGGAUUUUGUUCUCAGCAACUAGGGUGGAACCAGUUUAAAACCGGAUACGA<br>AAUCGUUCCGCCUAGUAUCUACCCUA

Name	Transcripts (5'→3')
eIT03	GGGGCAGUGAGAGAAAGAAGUACUUGCGUUUACCUCAUGAAAGCGACCUUAGGGCG<br>GUGUAAGCUAAGGAUGAGCACGCAACGAAAGGCAUUCUUGAGCCCUUUUAAAAAAG<br>AGGCUGGGAUUUUGUUCUCAGCAACUAGGGUGGAACCAGUAUUGAAAAAGCCAAAC<br>UAGGGUCUGUGGUUUCGCCAUCCCUA
eIT04	GGGGCAGUGAGAGAAAGAAGUACUUGCGUUUACCUCAUGAAAGCGACCUUAGGGCG<br>GUGUAAGCUAAGGAUGAGCACGCAACGAAAGGCAUUCUUGAGCCCUUUUAAAAAAG<br>AGGCUGGGAUUUUGUUCUCAGCAACUAGGGUGGAACCGAAUCCAUCUAUGCAUCAC<br>ACCAUAGAUGCCCGGUUGACACCCUA
eIT05	GGGGCAGUGAGAGAAAGAAGUACUUGCGUUUACCUCAUGAAAGCGACCUUAGGGCG<br>GUGUAAGCUAAGGAUGAGCACGCAACGAAAGGCAUUCUUGAGCCCUUUUAAAAAAG<br>AGGCUGGGAUUUUGUUCUCAGCAACUAGGGUGGAACCUAGAACCCGUCAGCGCCUC<br>GCCGAUGCUCUGGGAAGCCAUCCCUA
eIT06	GGGGCAGTGAGAGAAAGAAGTACTTGCGTTTACCTCATGAAAGCGACCTTAGGGCG<br>GTGTAAGCTAAGGATGAGCACGCAACGAAAGGCATTCTTGAGCCCTTTTAAAAAAG<br>AGGCTGGGATTTTGTTCTCAGCAACTAGGGTGGAACCGAATCCCTAGTTATGCAGA<br>TCAAGCTAGGCTCGGTTGGCACCCTA
eIT07	GGGGCAGUGAGAGAAAGAAGUACUUGCGUUUACCUCAUGAAAGCGACCUUAGGGCG<br>GUGUAAGCUAAGGAUGAGCACGCAACGAAAGGCAUUCUUGAGCCCUUUUAAAAAAG<br>AGGCUGGGAUUUUGUUCUCAGCAACUAGGGUGGAACCGAUAACCGCGCACUAACUA<br>AGUGGUAGCGCCAACCUUCUACCCUA
eIT08	GGGGCAGUGAGAGAAAGAAGUACUUGCGUUUACCUCAUGAAAGCGACCUUAGGGCG<br>GUGUAAGCUAAGGAUGAGCACGCAACGAAAGGCAUUCUUGAGCCCUUUUAAAAAAG<br>AGGCUGGGAUUUUGUUCUCAGCAACUAGGGUGGAACCAAGCCCGGCGUACCGCAUG<br>UCUGCGCUGCCUAGUUCAACACCCUA
eIT09	GGGGCAGUGAGAGAAAGAAGUACUUGCGUUUACCUCAUGAAAGCGACCUUAGGGCG<br>GUGUAAGCUAAGGAUGAGCACGCAACGAAAGGCAUUCUUGAGCCCUUUUAAAAAAG<br>AGGCUGGGAUUUUGUUCUCAGCAACUAGGGUGGAACCCGCCCUUGAAUGUGGAGCC<br>AAGGCCCAGCAAUUAUCCACGCCCUA
eIT10	GGGGCAGUGAGAGAAAGAAGUACUUGCGUUUACCUCAUGAAAGCGACCUUAGGGCG<br>GUGUAAGCUAAGGAUGAGCACGCAACGAAAGGCAUUCUUGAGCCCUUUUAAAAAAG<br>AGGCUGGGAUUUUGUUCUCAGCAACUAGGGUGGAACCAGGUAAGGUUGCGCCACCA<br>AGCAACUAUUGGUGUCCACCACCCUA

Name	Transcripts (5'→3')
eIT11	GGGGCAGUGAGAGAAAGAAGUACUUGCGUUUACCUCAUGAAAGCGACCUUAGGGCG<br>GUGUAAGCUAAGGAUGAGCACGCAACGAAAGGCAUUCUUGAGCCCUUUUAAAAAAG<br>AGGCUGGGAUUUUGUUCUCAGCAACUAGGGUGGAACCAGACCCGGACAGAGGAAGA<br>CCUCAGUUCGCCUGGUCCACACCCUA
eIT12	GGGGCAGUGAGAGAAAGAAGUACUUGCGUUUACCUCAUGAAAGCGACCUUAGGGCG<br>GUGUAAGCUAAGGAUGAGCACGCAACGAAAGGCAUUCUUGAGCCCUUUUAAAAAAG<br>AGGCUGGGAUUUUGUUCUCAGCAACUAGGGUGGAACCAGCAAGAAAGCCGACUAAC<br>GGACCUCGAACACAACAACCACCCUA
eIT13	GGGGCAGUGAGAGAAAGAAGUACUUGCGUUUACCUCAUGAAAGCGACCUUAGGGCG<br>GUGUAAGCUAAGGAUGAGCACGCAACGAAAGGCAUUCUUGAGCCCUUUUAAAAAAG<br>AGGCUGGGAUUUUGUUCUCAGCAACUAGGGUGGAACCCGAAGAGGGUUUUCAGGGA<br>GUUAAUUUGGUUUCGUCACCUCCCUA
eIT14	GGGGCAGUGAGAGAAAGAAGUACUUGCGUUUACCUCAUGAAAGCGACCUUAGGGCG<br>GUGUAAGCUAAGGAUGAGCACGCAACGAAAGGCAUUCUUGAGCCCUUUUAAAAAAG<br>AGGCUGGGAUUUUGUUCUCAGCAACUAGGGUGGAACCACCAGACUAACACCAUAGG<br>GAGGAAGUGGAUCCGUCACCUCCCUA
eIT15	GGGGCAGUGAGAGAAAGAAGUACUUGCGUUUACCUCAUGAAAGCGACCUUAGGGCG<br>GUGUAAGCUAAGGAUGAGCACGCAACGAAAGGCAUUCUUGAGCCCUUUUAAAAAAG<br>AGGCUGGGAUUUUGUUCUCAGCAACUAGGGUGGAACCGUUCCUAGAUAACUAACAA<br>GAAUAUUCCAAGCCAUAAACACCCUA
eIT16	GGGGCAGUGAGAGAAAGAAGUACUUGCGUUUACCUCAUGAAAGCGACCUUAGGGCG<br>GUGUAAGCUAAGGAUGAGCACGCAACGAAAGGCAUUCUUGAGCCCUUUUAAAAAAG<br>AGGCUGGGAUUUUGUUCUCAGCAACUAGGGUGGAACCAGACCCCAUCCAGUAUCAC<br>CAAACUGGAUGGCCUAUAACACCCUA
eIT17	GGGGCAGUGAGAGAAAGAAGUACUUGCGUUUACCUCAUGAAAGCGACCUUAGGGCG<br>GUGUAAGCUAAGGAUGAGCACGCAACGAAAGGCAUUCUUGAGCCCUUUUAAAAAAG<br>AGGCUGGGAUUUUGUUCUCAGCAACUAGGGUGGAACCAGUUUAAAUCCCUUCGCAG<br>UCGCGGAGGCCUGAGCUUUCACCCUA
eIT18	GGGGCAGUGAGAGAAAGAAGUACUUGCGUUUACCUCAUGAAAGCGACCUUAGGGCG<br>GUGUAAGCUAAGGAUGAGCACGCAACGAAAGGCAUUCUUGAGCCCUUUUAAAAAAG<br>AGGCUGGGAUUUUGUUCUCAGCAACUAGGGUGGAACCGAACCCGGUCACCACACCG<br>UGGAAAAUCGCUCGGUUACUACCCUA

Name	Transcripts (5'→3')
eIT19	GGGGCAGUGAGAGAAAGAAGUACUUGCGUUUACCUCAUGAAAGCGACCUUAGGGCG<br>GUGUAAGCUAAGGAUGAGCACGCAACGAAAGGCAUUCUUGAGCCCUUUUAAAAAAG<br>AGGCUGGGAUUUUGUUCUCAGCAACUAGGGUGGAACCAGAUCCCCUCCCUCAUUCG<br>CAAGGUAGGGCCUGAUUCGCACCCUA
eIT20	GGGGCAGUGAGAGAAAGAAGUACUUGCGUUUACCUCAUGAAAGCGACCUUAGGGCG<br>GUGUAAGCUAAGGAUGAGCACGCAACGAAAGGCAUUCUUGAGCCCUUUUAAAAAAG<br>AGGCUGGGAUUUUGUUCUCAGCAACUAGGGUGGAACCGCAUGUUUUGCCUCUAUCG<br>GCCAAGAAUAUCCCGGAUGCACCCUA
eIT21	GGGGCAGUGAGAGAAAGAAGUACUUGCGUUUACCUCAUGAAAGCGACCUUAGGGCG<br>GUGUAAGCUAAGGAUGAGCACGCAACGAAAGGCAUUCUUGAGCCCUUUUAAAAAAG<br>AGGCUGGGAUUUUGUUCUCAGCAACUAGGGUGGAACCUGGCUUCCCCAUCCACCCU<br>AACCUAAAUCGAGGUACCCAACCCUA
eIT22	GGGGCAGUGAGAGAAAGAAGUACUUGCGUUUACCUCAUGAAAGCGACCUUAGGGCG<br>GUGUAAGCUAAGGAUGAGCACGCAACGAAAGGCAUUCUUGAGCCCUUUUAAAAAAG<br>AGGCUGGGAUUUUGUUCUCAGCAACUAGGGUGGAACCGAACCCGCUUGUCUCAAUG<br>UGGCUAGCGCUCGGUUCAACACCCUA
eIT23	GGGGCAGUGAGAGAAAGAAGUACUUGCGUUUACCUCAUGAAAGCGACCUUAGGGCG<br>GUGUAAGCUAAGGAUGAGCACGCAACGAAAGGCAUUCUUGAGCCCUUUUAAAAAAG<br>AGGCUGGGAUUUUGUUCUCAGCAACUAGGGUGGAACCCCGUGAACAGAGGAAGGGG<br>AGUUUUUGGGUCUGCCAACUACCCUA
eIT24	GGGGCAGUGAGAGAAAGAAGUACUUGCGUUUACCUCAUGAAAGCGACCUUAGGGCG<br>GUGUAAGCUAAGGAUGAGCACGCAACGAAAGGCAUUCUUGAGCCCUUUUAAAAAAG<br>AGGCUGGGAUUUUGUUCUCAGCAACUAGGGUGGAACCGAAUCCGUCUAUCUCGAUA<br>AUGGCAGACGCCCGGUUUCUACCCUA
eIT25	GGGGCAGUGAGAGAAAGAAGUACUUGCGUUUACCUCAUGAAAGCGACCUUAGGGCG<br>GUGUAAGCUAAGGAUGAGCACGCAACGAAAGGCAUUCUUGAGCCCUUUUAAAAAAG<br>AGGCUGGGAUUUUGUUCUCAGCAACUAGGGUGGAACCGCACCCGUAGGGUACAUGC<br>CGGCCCUAUGCGCGAUUACUACCCUA
eIT26	GGGGCAGUGAGAGAAAGAAGUACUUGCGUUUACCUCAUGAAAGCGACCUUAGGGCG<br>GUGUAAGCUAAGGAUGAGCACGCAACGAAAGGCAUUCUUGAGCCCUUUUAAAAAAG<br>AGGCUGGGAUUUUGUUCUCAGCAACUAGGGUGGAACCAGCGAGCAUCCGAAUAAGG<br>UUGUACUCAGACUUCCAACCACCCUA

Name	Transcripts (5'→3')
eIT27	GGGGCAGUGAGAGAAAGAAGUACUUGCGUUUACCUCAUGAAAGCGACCUUAGGGCG<br>GUGUAAGCUAAGGAUGAGCACGCAACGAAAGGCAUUCUUGAGCCCUUUUAAAAAAG<br>AGGCUGGGAUUUUGUUCUCAGCAACUAGGGUGGAACCGAAUCCGUAGCCGGUUUGC<br>CGUGCAUACGCCCGGUUGGCACCCUA
eIT28	GGGGCAGUGAGAGAAAGAAGUACUUGCGUUUACCUCAUGAAAGCGACCUUAGGGCG<br>GUGUAAGCUAAGGAUGAGCACGCAACGAAAGGCAUUCUUGAGCCCUUUUAAAAAAG<br>AGGCUGGGAUUUUGUUCUCAGCAACUAGGGUGGAACCAUUUGGUUUCGCGGGUAGG<br>GGAUAGGUCCCUUCCAAACCACCCUA
eIT29	GGGGCAGUGAGAGAAAGAAGUACUUGCGUUUACCUCAUGAAAGCGACCUUAGGGCG<br>GUGUAAGCUAAGGAUGAGCACGCAACGAAAGGCAUUCUUGAGCCCUUUUAAAAAAG<br>AGGCUGGGAUUUUGUUCUCAGCAACUAGGGUGGAACCGAAUCCCCGGAGGAAAUAU<br>CCCGAACCGGGCCCGACUCUACCCUA
eIT30	GGGGCAGUGAGAGAAAGAAGUACUUGCGUUUACCUCAUGAAAGCGACCUUAGGGCG<br>GUGUAAGCUAAGGAUGAGCACGCAACGAAAGGCAUUCUUGAGCCCUUUUAAAAAAG<br>AGGCUGGGAUUUUGUUCUCAGCAACUAGGGUGGAACCAAAUCCACCAGGCCACAAA<br>CUGCGAUUUAAAACCUUGUGACCCUA
eIT31	GGGGCAGUGAGAGAAAGAAGUACUUGCGUUUACCUCAUGAAAGCGACCUUAGGGCG<br>GUGUAAGCUAAGGAUGAGCACGCAACGAAAGGCAUUCUUGAGCCCUUUUAAAAAAG<br>AGGCUGGGAUUUUGUUCUCAGCAACUAGGGUGGAACCCCAGAAGGCCCGCCCUUGA<br>AUGUGGAGCAAUGAACGAGAACCCUA
eIT32	GGGGCAGUGAGAGAAAGAAGUACUUGCGUUUACCUCAUGAAAGCGACCUUAGGGCG<br>GUGUAAGCUAAGGAUGAGCACGCAACGAAAGGCAUUCUUGAGCCCUUUUAAAAAAG<br>AGGCUGGGAUUUUGUUCUCAGCAACUAGGGUGGAACCGUCACAGUACGCCCACCCC<br>AAGAACUUCUAGAACUAAUCCCCCUA
eIT33	GGGGCAGUGAGAGAAAGAAGUACUUGCGUUUACCUCAUGAAAGCGACCUUAGGGCG<br>GUGUAAGCUAAGGAUGAGCACGCAACGAAAGGCAUUCUUGAGCCCUUUUAAAAAAG<br>AGGCUGGGAUUUUGUUCUCAGCAACUAGGGUGGAACCAGCAUUGAUUCUCAGAAUC<br>UACGAAGCUACAAAACAACCACCCUA
eIT34	GGGGCAGUGAGAGAAAGAAGUACUUGCGUUUACCUCAUGAAAGCGACCUUAGGGCG<br>GUGUAAGCUAAGGAUGAGCACGCAACGAAAGGCAUUCUUGAGCCCUUUUAAAAAAG<br>AGGCUGGGAUUUUGUUCUCAGCAACUAGGGUGGAACCUCCUCCUACGAUGGUUAGC<br>UCCAUCGCAAGCGAAUAUCUACCCUA

Name	Transcripts (5'→3')
eIT35	GGGGCAGUGAGAGAAAGAAGUACUUGCGUUUACCUCAUGAAAGCGACCUUAGGGCG<br>GUGUAAGCUAAGGAUGAGCACGCAACGAAAGGCAUUCUUGAGCCCUUUUAAAAAAG<br>AGGCUGGGAUUUUGUUCUCAGCAACUAGGGUGGAACCAAAGCAAAAAAAAGGGAAG<br>UUUUUGGUUCUGCCAACUGUACCCUA
eIT36	GGGGCAGUGAGAGAAAGAAGUACUUGCGUUUACCUCAUGAAAGCGACCUUAGGGCG<br>GUGUAAGCUAAGGAUGAGCACGCAACGAAAGGCAUUCUUGAGCCCUUUUAAAAAAG<br>AGGCUGGGAUUUUGUUCUCAGCAACUAGGGUGGAACCUCGCUGAGACAAAUUUAGG<br>GCUGAAAUUGGAUUCCCCAUACCCUA
eIT32$_{CCC}$	GGGGCAGUGAGAGAAAGAAGUACUUGCGUUUACCUCAUGAAAGCGACCUUAGGGCG<br>GUGUAAGCUAAGGAUGAGCACGCAACGAAACCCAUUCUUGAGCCCUUUUAAAAAAG<br>AGGCUGGGAUUUUGUUCUCAGCAACUAGGGUGGAACCGUCACAGUACGCCCACCCC<br>AAGAACUUCUAGAACUAAUCCCCCUA
eIT32$_{CCG}$	GGGGCAGUGAGAGAAAGAAGUACUUGCGUUUACCUCAUGAAAGCGACCUUAGGGCG<br>GUGUAAGCUAAGGAUGAGCACGCAACGAAACCGAUUCUUGAGCCCUUUUAAAAAAG<br>AGGCUGGGAUUUUGUUCUCAGCAACUAGGGUGGAACCGUCACAGUACGCCCACCCC<br>AAGAACUUCUAGAACUAAUCCCCCUA
eIT32$_{CGC}$	GGGGCAGUGAGAGAAAGAAGUACUUGCGUUUACCUCAUGAAAGCGACCUUAGGGCG<br>GUGUAAGCUAAGGAUGAGCACGCAACGAAACGCAUUCUUGAGCCCUUUUAAAAAAG<br>AGGCUGGGAUUUUGUUCUCAGCAACUAGGGUGGAACCGUCACAGUACGCCCACCCC<br>AAGAACUUCUAGAACUAAUCCCCCUA
eIT32$_{CGG}$	GGGGCAGUGAGAGAAAGAAGUACUUGCGUUUACCUCAUGAAAGCGACCUUAGGGCG<br>GUGUAAGCUAAGGAUGAGCACGCAACGAAACGGAUUCUUGAGCCCUUUUAAAAAAG<br>AGGCUGGGAUUUUGUUCUCAGCAACUAGGGUGGAACCGUCACAGUACGCCCACCCC<br>AAGAACUUCUAGAACUAAUCCCCCUA
eIT32$_{GCC}$	GGGGCAGUGAGAGAAAGAAGUACUUGCGUUUACCUCAUGAAAGCGACCUUAGGGCG<br>GUGUAAGCUAAGGAUGAGCACGCAACGAAAGCCAUUCUUGAGCCCUUUUAAAAAAG<br>AGGCUGGGAUUUUGUUCUCAGCAACUAGGGUGGAACCGUCACAGUACGCCCACCCC<br>AAGAACUUCUAGAACUAAUCCCCCUA
eIT32$_{GCG}$	GGGGCAGUGAGAGAAAGAAGUACUUGCGUUUACCUCAUGAAAGCGACCUUAGGGCG<br>GUGUAAGCUAAGGAUGAGCACGCAACGAAAGCGAUUCUUGAGCCCUUUUAAAAAAG<br>AGGCUGGGAUUUUGUUCUCAGCAACUAGGGUGGAACCGUCACAGUACGCCCACCCC<br>AAGAACUUCUAGAACUAAUCCCCCUA

Name	Transcripts (5'→3')
eIT32$_{GGG}$	GGGGCAGUGAGAGAAAGAAGUACUUGCGUUUACCUCAUGAAAGCGACCUUAGGGCGGUGUAAGCUAAGGAUGAGCACGCAACGAAAGGGAUUCUUGAGCCCUUUUAAAAAAGAGGCUGGGAUUUUGUUCUCAGCAACUAGGGUGGAACCGUCACAGUACGCCCACCCCAAGAACUUCUAGAACUAAUCCCCCUA
eIT32$_{UUA}$	GGGGCAGUGAGAGAAAGAAGUACUUGCGUUUACCUCAUGAAAGCGACCUUAGGGCGGUGUAAGCUAAGGAUGAGCACGCAACGAAAUUAAUUCUUGAGCCCUUUUAAAAAAGAGGCUGGGAUUUUGUUCUCAGCAACUAGGGUGGAACCGUCACAGUACGCCCACCCCAAGAACUUCUAGAACUAAUCCCCCUA
Library02	GGGGCAGUGAGAGAAAGAAGUACUUGCGUUUACCUCAUGAAAGCGACCUUAGGGCGGUGUAAGCUAAGGAUGAGCACGCAACGAAAGCCAUUAUUGAGCCCUUUUAAAAAAGAGGCUGGGAUUUUGUUCUCAGCAACUAGGGUGGAACCGU(NNNNN)$_{5-7}$ACG(N)$_{1-3}$CCACCCCAAGAACUUCUAGAACUAAUCCCCCUA
Library03	GGGGCAGUGAGAGAAAGAAGUACUUGCGUUUACCUCAUGAAAGCGACCUUAGGGCGGUGUAAGCUAAGGAUGAGCACGCAACGAAAGCCAUUAUUGAGCCCUUUUAAAAAAGAGGCUGGGAUUUUGUUCUCAGCAACUAGGGUGGAACCGUCACAGUACGCCCACCCNNNNNNNNNNNNNNNNNNNNNNCCCUA
eIT37	GGGGCAGUGAGAGAAAGAAGUACUUGCGUUUACCUCAUGAAAGCGACCUUAGGGCGGUGUAAGCUAAGGAUGAGCACGCAACGAAAGCCAUUAUUGAGCCCUUUUAAAAAAGAGGCUGGGAUUUUGUUCUCAGCAACUAGGGUGGAACCGUCACAGUACGCCCACCCCAAGAACUUCUAGACCUAAUCCCCCUA
eIT38	GGGGCAGUGAGAGAAAGAAGUACUUGCGUUUACCUCAUGAAAGCGACCUUAGGGCGGUGUAAGCUAAGGAUGAGCACGCAACGAAAGCCAUUAUUGAGCCCUUUUAAAAAAGAGGCUGGGAUUUUGUUCUCAGCAACUAGGGUGGAACCGUCACAGUACGCCCACCCCAAGAACUUCUAGAGCUAAUCCCCCUA
eIT39	GGGGCAGUGAGAGAAAGAAGUACUUGCGUUUACCUCAUGAAAGCGACCUUAGGGCGGUGUAAGCUAAGGAUGAGCACGCAACGAAAGCCAUUAUUGAGCCCUUUUAAAAAAGAGGCUGGGAUUUUGUUCUCAGCAACUAGGGUGGAACCGUCACAGUACGCCCACCCCAAGAACUUCUAGAACUAACCCCCCUA
eIT40	GGGGCAGUGAGAGAAAGAAGUACUUGCGUUUACCUCAUGAAAGCGACCUUAGGGCGGUGUAAGCUAAGGAUGAGCACGCAACGAAAGCCAUUAUUGAGCCCUUUUAAAAAAGAGGCUGGGAUUUUGUUCUCAGCAACUAGGGUGGAACCGUCACAGUACGCCCACCCCCAAGAACUUCUAGAACUAAUCCCCCUA

Name	Transcripts (5'→3')
eIT41	GGGGCAGUGAGAGAAAGAAGUACUUGCGUUUACCUCAUGAAAGCGACCUUAGGGCG<br>GUGUAAGCUAAGGAUGAGCACGCAACGAAAGCCAUUAUUGAGCCCUUUUAAAAAAG<br>AGGCUGGGAUUUUGUUCUCAGCAACUAGGGUGGAACCGUCACAGUACGCCCACCCC<br>AAGAACUUCUAGAACUAAUCCCCUA
eIT42	GGGGCAGUGAGAGAAAGAAGUACUUGCGUUUACCUCAUGAAAGCGACCUUAGGGCG<br>GUGUAAGCUAAGGAUGAGCACGCAACGAAAGCCAUUAUUGAGCCCUUUUAAAAAAG<br>AGGCUGGGAUUUUGUUCUCAGCAACUAGGGUGGAACCGUCACAGUACCCCCACCCC<br>AAGAACUUCUAGAACUAAUCCCCCUA
eIT43	GGGGCAGUGAGAGAAAGAAGUACUUGCGUUUACCUCAUGAAAGCGACCUUAGGGCG<br>GUGUAAGCUAAGGAUGAGCACGCAACGAAAGCCAUUAUUGAGCCCUUUUAAAAAAG<br>AGGCUGGGAUUUUGUUCUCAGCAACUAGGGUGGAACCGUCACAGUACGCCCACCCC<br>AAGAACUUCCAGAACUAAUCCCCCUA
eIT44	GGGGCAGUGAGAGAAAGAAGUACUUGCGUUUACCUCAUGAAAGCGACCUUAGGGCG<br>GUGUAAGCUAAGGAUGAGCACGCAACGAAAGCCAUUAUUGAGCCCUUUUAAAAAAG<br>AGGCUGGGAUUUUGUUCUCAGCAACUAGGGUGGAACCGUCACAGUACGCCCGCCCC<br>AAGAACUUCUAGAACUAAUCCCCCUA
eIT45	GGGGCAGUGAGAGAAAGAAGUACUUGCGUUUACCUCAUGAAAGCGACCUUAGGGCG<br>GUGUAAGCUAAGGAUGAGCACGCAACGAAAGCCAUUAUUGAGCCCUUUUAAAAAAG<br>AGGCUGGGAUUUUGUUCUCAGCAACUAGGGUGGAACCGUCACAGCACGCCCACCCC<br>AAGAACUUCUAGAACUAAUCCCCCUA
eIT46	GGGGCAGUGAGAGAAAGAAGUACUUGCGUUUACCUCAUGAAAGCGACCUUAGGGCG<br>GUGUAAGCUAAGGAUGAGCACGCAACGAAAGCCAUUAUUGAGCCCUUUUAAAAAAG<br>AGGCUGGGAUUUUGUUCUCAGCAACUAGGGUGGAACCGUCACAGUACGCCCACCCC<br>AAGAACCUCUAGAACUAAUCCCCCUA
eIT47	GGGGCAGUGAGAGAAAGAAGUACUUGCGUUUACCUCAUGAAAGCGACCUUAGGGCG<br>GUGUAAGCUAAGGAUGAGCACGCAACGAAAGCCAUUAUUGAGCCCUUUUAAAAAAG<br>AGGCUGGGAUUUUGUUCUCAGCAACUAGGGUGGAACCGUCACGGUACGCCCACCCC<br>AAGAACUUCUAGAACUAAUCCCCCUA
eIT48	GGGGCAGUGAGAGAAAGAAGUACUUGCGUUUACCUCAUGAAAGCGACCUUAGGGCG<br>GUGUAAGCUAAGGAUGAGCACGCAACGAAAGCCAUUAUUGAGCCCUUUUAAAAAAG<br>AGGCUGGGAUUUUGUUCUCAGCAACUAGGGUGGAACCGUCACAGUACGCCCACCCC<br>AAGAACUUCUAGAACUCAUCCCCCUA

Name	Transcripts (5'→3')
eIT49	GGGGCAGUGAGAGAAAGAAGUACUUGCGUUUACCUCAUGAAAGCGACCUUAGGGCG<br>GUGUAAGCUAAGGAUGAGCACGCAACGAAAGCCAUUAUUGAGCCCUUUUAAAAAAG<br>AGGCUGGGAUUUUGUUCUCAGCAACUAGGGUGGAACCGUCACAGUACGCCCACCCC<br>CAAGAACUUCUAGAACUAAUCCCCUA
tRNA$^{Gly}_{GGG}$	GCGGAAGUAGUUCAGUGGUAGAACACCACCUUGGGAAGGUGGGGGUCGCGGGUUCG<br>AAUCCCGUCUUCCGCUCCA
tRNA$^{Gly}_{CGC}$	GCGGAAGUAGUUCAGUGGUAGAACACCACCUUCGGAAGGUGGGGGUCGCGGGUUCG<br>AAUCCCGUCUUCCGCUCCA
tRNA$^{Gly}_{GCG}$	GCGGAAGUAGUUCAGUGGUAGAACACCACCUUGCGAAGGUGGGGGUCGCGGGUUCG<br>AAUCCCGUCUUCCGCUCCA
tRNA$^{Gly}_{CCG}$	GCGGAAGUAGUUCAGUGGUAGAACACCACCUUCCGAAGGUGGGGGUCGCGGGUUCG<br>AAUCCCGUCUUCCGCUCCA
tRNA$^{Gly}_{GGC}$	GCGGAAGUAGUUCAGUGGUAGAACACCACCUUGCCAAGGUGGGGGUCGCGGGUUCG<br>AAUCCCGUCUUCCGCUCCA
tRNA$^{Gly}_{CGC}$	GCGGAAGUAGUUCAGUGGUAGAACACCACCUUCGCAAGGUGGGGGUCGCGGGUUCG<br>AAUCCCGUCUUCCGCUCCA
tRNA$^{Gly}_{GCC}$	GCGGAAGUAGUUCAGUGGUAGAACACCACCUUGCCAAGGUGGGGGUCGCGGGUUCG<br>AAUCCCGUCUUCCGCUCCA
tRNA$^{Gly}_{CCC}$	GCGGAAGUAGUUCAGUGGUAGAACACCACCUUCCCAAGGUGGGGGUCGCGGGUUCG<br>AAUCCCGUCUUCCGCUCCA
tRNA$^{Gly}_{UAA}$	GCGGAAGUAGUUCAGUGGUAGAACACCACCUUUAAAAGGUGGGGGUCGCGGGUUCG<br>AAUCCCGUCUUCCGCUCCA
tRNA$^{Gly}_{GCA}$	GCGGAAGUAGUUCAGUGGUAGAACACCACCUUGCAAAGGUGGGGGUCGCGGGUUCG<br>AAUCCCGUCUUCCGCUCCA
tRNA$^{Gly}_{GAC}$	GCGGAAGUAGUUCAGUGGUAGAACACCACCUUGACAAGGUGGGGGUCGCGGGUUCG<br>AAUCCCGUCUUCCGCUCCA
tRNA$^{Gly}_{UCC}$	GCGGAAGUAGUUCAGUGGUAGAACACCACCUUUCCAAGGUGGGGGUCGCGGGUUCG<br>AAUCCCGUCUUCCGCUCCA
tRNA$^{Gly}_{GAA}$	GCGGAAGUAGUUCAGUGGUAGAACACCACCUUGAAAAGGUGGGGGUCGCGGGUUCG<br>AAUCCCGUCUUCCGCUCCA

Name	Transcripts (5'→3')
$tRNA^{Gly}_{UCA}$	GCGGAAGUAGUUCAGUGGUAGAACACCACCUUUCAAAGGUGGGGGUCGCGGGUUCG AAUCCCGUCUUCCGCUCCA
$tRNA^{Gly}_{UAC}$	GCGGAAGUAGUUCAGUGGUAGAACACCACCUUUACAAGGUGGGGGUCGCGGGUUCG AAUCCCGUCUUCCGCUCCA
mR1	GGGUUAACUUUAAGAAGGAGAUAUACAUAUGAAGAAAAAGCGCGAUUACAAAGACG ACGACGACAAGUAAGACGGGGGGCGGAAA
mR2	GGGUUAACUUUAAGAAGGAGAUAUACAUAUGAAGAAAAAGGCCGAUUACAAAGACG ACGACGACAAGUAAGACGGGGGGCGGAAA
mR3	GGGUUAACUUUAAGAAGGAGAUAUACAUAUGAAGAAAAAGGGCGAUUACAAAGACG ACGACGACAAGUAAGACGGGGGGCGGAAA

N denotes for all possible nucleotides.

Supplementary Table 2 | Primers

Name	Sequences (5'→3')	Used for
Oligo1	GGCGTAATACGACTCACTATAGGGGCAGTGAGAGAAAGAAGTACTTGCGTTTACCTCATGAAAG	Library construction common
Oligo2	GTACTTGCGTTTACCTCATGAAAGCGACCTTAGGGCGGTGTAAGCTAAGGATGAGCACGCAACGAAAGGCATTCTTGAGCCCTTTTAAAAAAGAGGCTGGGATTTTGTTCTCAGCAACTAGGGTGGAACC	Library01 construction
Oligo3	TGGAGCGGAAGACGGGATTCGAACCCGCGACCCCCACCTTGGCAAGGTGGTGTTCTACCACTGAACTACTTCCGCTTTTTTTTTTTTTTTTAGGGNNNNNNNNNNNNNNNNNNNNNNNNNNNNNNNNNNNNNNNNNNGGTTCCACCCTAGTTGCTGA	Library01 construction
Oligo4	TGmGAGCGGAAGACGGGA	Library construction common, RT, PCR amplification, and tRNA
Oligo5	GGCGTAATACGACTCACTATAGGGGCAGTGAGAGAAAGAA	PCR amplification
Oligo6	AAATGGAGCGGAAGACGGGATTCGAACCCGCGACC	b-tREX
Oligo7	ACACTCTTTCCCTACACGACGCTCTTCCGATCTTCAGCAACTAGGGTGGAACC	Sequencing
Oligo8	GTGACTGGAGTTCAGACGTGTGCTCTTCCGATCTTACCACTGAACTACTTCCGC	Sequencing
Oligo9	AATGATACGGCGACCACCGAGATCTACACNNNNNNNNACACTCTTTCCCTACACGAC	Sequencing
Oligo10	CAAGCAGAAGACGGCATACGAGATNNNNNNNNGTGACTGGAGTTCAGACGTG	Sequencing
Oligo11	GGCGTAATACGACTCACTATAGGGGCAGTGAGAGAAAGAAGTACTTGCGTTTACCTCATGAAAGCGACCT	Clone preparation common
Oligo12	CCACCCTAGTTGCTGAGAACAAAATCCCAGCCTCTTTTTTAAAAGGGCTCAAGAATGCCTTTCGTTGCGT	Clone preparation common
Oligo13	TTTACCTCATGAAAGCGACCTTAGGGCGGTGTAAGCTAAGGATGAGCACGCAACGAAAGGCATTCTTGA	Clone preparation common
Oligo14	TAGGGATGTCCGTGCGATCATGCTTTTATTTCATTAACAGATTCAGGTTCCACCCTAGTTGCTGAGAACAA	inT1-tRNA, inT1
Oligo15	ATGATCGCACGGACATCCCTAAAAAAAAAAAAAAAAGCGGAAGTAGTTCAGTGGTA	inT1-tRNA

Name	Sequences (5'→3')	Used for
Oligo16	GGCGTAATACGACTCACTATAGGGTAGGGTGGAACCTGAATCTG	inT1-at
Oligo17	GTGCGATCATGCTTTTATTTCATTAACAGATTCAGGTTCCACCC	inT1-at
Oligo18	AGGGATGTCCGTGCGATCATGCTTTTATTT	inT1-at
Oligo19	GGCGTAATACGACTCACTATAGGCCTTCGGGCCAATAGGGTGGAACC	inT1-at-s
Oligo20	GAACCGGACCGAAGCCCGATTTGGATCCGGCGAACCGGATCGA	inT1-at-s
Oligo21	CGGGCCAATAGGGTGGAACCTGAATCTGTTAATGAAATAAAAGCATGAT	inT1-at-s
Oligo22	GATCCGGCGAACCGGATCGAATGGGATGTCCGTGCGATCATGCTTTTATTTCATTAA	inT1-at-s
Oligo23	GAACCGGACCGAAGCCCG	Primer extension
Oligo24	TAGGGCGTGGATAATTGCTGGGCCTTGGCTCCACATTCAAGGGCGGGTTCCACCCTAGTTGCTGAGAACA	eIT09
Oligo25	TAGGGTTCTCGTTCATTGCTCCACATTCAAGGGCGGGCCTTCTGGGGTTCCACCCTAGTTGCTGAGAACA	eIT31
Oligo26	TAGGGGGATTAGTTCTAGAAGTTCTTGGGGTGGGCGTACTGTGACGGTTCCACCCTAGTTGCTGAGAACA	eIT32
Oligo27	CACCCTAGTTGCTGAGAACAAAATCCCAGCCTCTTTTTTAAAAGGGCTCAAGAATGGCTTTCGTTGCGTG	eIT32$_{GCC}$
Oligo28	TAGGGGGATTAGTTCTAGAAGTTCTTGGGGTGGGCGTACTGTGACGGTTCCACCCTAGTTGCTGAGAACA	eIT32$_{GCC}$
Oligo29	TTACCTCATGAAAGCGACCTTAGGGCGGTGTAAGCTAAGGATGAGCACGCAACGAAAGCCATTCTTGAGCCCTTTTAAAAAAGAGGCTGGGATTTTGTTCTCAGCAACTAGGGTGGAACCGT	Library02, 03 construction
Oligo30	TGGAGCGGAAGACGGGATTCGAACCCGCGACCCCCACCTTGGCAAGGTGGTGTTCTACCACTGAACTACTTCCGCTTTTTTTTTTTTTTTTAGGGGGATTAGTTCTAGAAGTTCTTGGGGTGG(N)$_{1-3}$CGT(NNNNN)$_{5-7}$ACGGTTCCACCCTAGTTGCT	Library02 construction

Name	Sequences (5'→3')	Used for
Oligo31	TGGAGCGGAAGACGGGATTCGAACCCGCGACCCCCACCTTGGCAAGGTGGTGTTCTACCACTGAACTACTTCCGCTTTTTTTTTTTTTTTTTAGGGNNNNNNNNNNNNNNNNNNNNNNGGGTGGGCGTACTGTGACGG	Library03 construction
Oligo32	TAGGGGGATTAGTTCTAGAAGTTCTTGGGGGTGGGCGTACTGTGACGGTTCCACCCTAGTTGCTGAGAAC	eIT40
Oligo33	TAGGGGATTAGTTCTAGAAGTTCTTGGGGTGGGCGTACTGTGACGGTTCCACCCTAGTTGCTGAGAACAA	eIT41
Oligo34	TAGGGGATTAGTTCTAGAAGTTCTTGGGGGTGGGCGTACTGTGACGGTTCCACCCTAGTTGCTGAGAACA	eIT49
Oligo35	TACCTAACGCTAATCCCCT	eFx
Oligo36	ACCTAACGCTAATCCCCTTTCGGGGCCGCGGAAATCTTTCGATCC	eFx
Oligo37	ACCTAACGCTAATCCCCT	eFx
Oligo38	GGCGTAATACGACTCACTATAG	eFx
Oligo39	GGCGTAATACGACTCACTATAGCGGAAGTAGTTCAGTGGTAGAACACCACCTT	$tRNA^{Gly}$ common
Oligo40	TGGAGCGGAAGACGGGATTCGAACCCGCGACCCCCACCTTCCCAAGGTGGTGTTCTACCACTG	$tRNA^{Gly}_{GGG}$
Oligo41	TGGAGCGGAAGACGGGATTCGAACCCGCGACCCCCACCTTCCGAAGGTGGTGTTCTACCACTG	$tRNA^{Gly}_{CGC}$
Oligo42	TGGAGCGGAAGACGGGATTCGAACCCGCGACCCCCACCTTCGCAAGGTGGTGTTCTACCACTG	$tRNA^{Gly}_{GCG}$
Oligo43	TGGAGCGGAAGACGGGATTCGAACCCGCGACCCCCACCTTCGGAAGGTGGTGTTCTACCACTG	$tRNA^{Gly}_{CCG}$
Oligo44	TGGAGCGGAAGACGGGATTCGAACCCGCGACCCCCACCTTGCCAAGGTGGTGTTCTACCACTG	$tRNA^{Gly}_{GGC}$
Oligo45	TGGAGCGGAAGACGGGATTCGAACCCGCGACCCCCACCTTGCGAAGGTGGTGTTCTACCACTG	$tRNA^{Gly}_{CGC}$

Name	Sequences (5'→3')	Used for
Oligo46	TGGAGCGGAAGACGGGATTCGAACCCGCGACCCCCACCTTGGCAAGGTGGTGTTCTACCACTG	$tRNA^{Gly}_{GCC}$
Oligo47	TGGAGCGGAAGACGGGATTCGAACCCGCGACCCCCACCTTGGGAAGGTGGTGTTCTACCACTG	$tRNA^{Gly}_{CCC}$
Oligo48	TGGAGCGGAAGACGGGATTCGAACCCGCGACCCCCACCTTTTAAAGGTGGTGTTCTACCACTG	$tRNA^{Gly}_{UAA}$
Oligo49	TGGAGCGGAAGACGGGATTCGAACCCGCGACCCCCACCTTTCCAAGGTGGTGTTCTACCACTG	$tRNA^{Gly}_{GGA}$
Oligo50	TGGAGCGGAAGACGGGATTCGAACCCGCGACCCCCACCTTGTCAAGGTGGTGTTCTACCACTG	$tRNA^{Gly}_{GAC}$
Oligo51	TGGAGCGGAAGACGGGATTCGAACCCGCGACCCCCACCTTGCAAAGGTGGTGTTCTACCACTG	$tRNA^{Gly}_{UGC}$
Oligo52	TGGAGCGGAAGACGGGATTCGAACCCGCGACCCCCACCTTTTCAAGGTGGTGTTCTACCACTG	$tRNA^{Gly}_{GAA}$
Oligo53	TGGAGCGGAAGACGGGATTCGAACCCGCGACCCCCACCTTTCAAAGGTGGTGTTCTACCACTG	$tRNA^{Gly}_{UGA}$
Oligo54	TGGAGCGGAAGACGGGATTCGAACCCGCGACCCCCACCTTGTAAAGGTGGTGTTCTACCACTG	$tRNA^{Gly}_{UAC}$
Oligo55	TAATACGACTCACTATAGGGTTAACTTTAAGAAGGAGATATACATATG	mR1, 2, 3
Oligo56	TTTCCGCCCCCCGTCTTACTTGTCGTCGTCGTCTTTG	mR1, 2, 3
Oligo57	GAAGGAGATATACATATGAAGAAAAAGCGCGATTA	mR1
Oligo58	TTGTCGTCGTCGTCTTTGTAATCGCGCTTTTTCTTC	mR1
Oligo59	GAAGGAGATATACATATGAAGAAAAAGGCCGATTA	mR2
Oligo60	TTGTCGTCGTCGTCTTTGTAATCGGCCTTTTTCTTC	mR2
Oligo61	GAAGGAGATATACATATGAAGAAAAAGGGCGATTA	mR3
Oligo62	TTGTCGTCGTCGTCTTTGTAATCGCCCTTTTTCTTC	mR3

Gm indicates 2′-O-methylated guanosine. N denotes for all possible nucleotides.

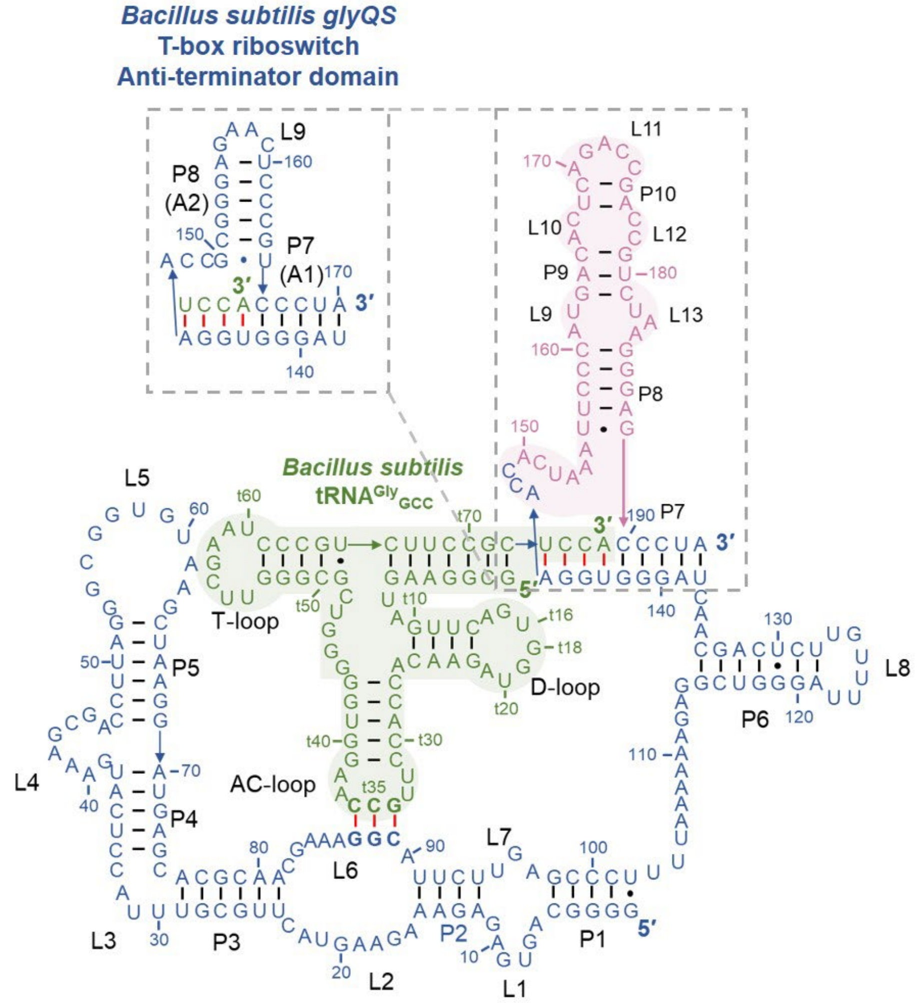

Supplementary Figure 1│Predicted secondary structure of Tx2.1 with its cognate tRNA^{Gly}$_{GCC}$. In previous work (50), we randomized the anti-terminator domain of *B. subtilis glyQS* T-box riboswitch into a 40-nt randomized library and obtained a T-boxzyme, Tx2.1, through *in vitro* selection. Blue letters indicate the T-box riboswitch scaffold, and green ones represent its cognate tRNA. The nucleotides derived from the 40-nt randomized library are colored in pink. Base pairs between T-box and tRNA are colored in red. The ‘t’ placed before numbers stands for tRNA numbering.

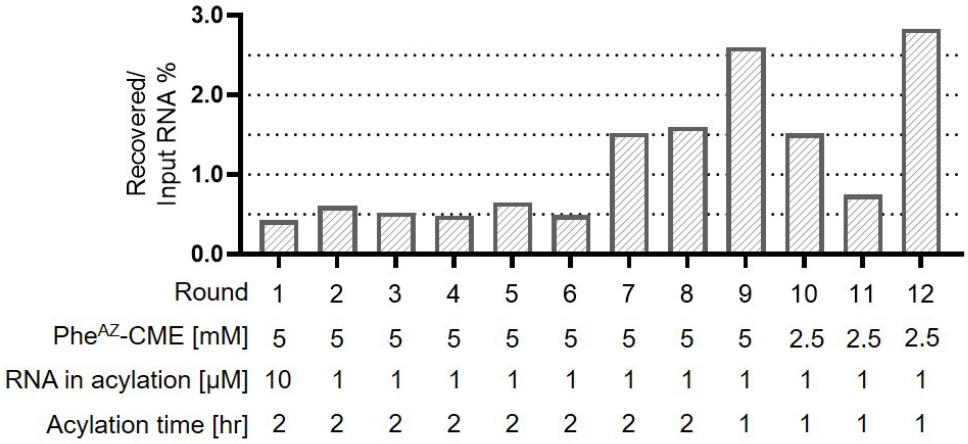

Supplementary Figure 2 | The process of click reaction-based *in vitro* selection. The ratio of RNA recovered from SAv-immobilized magnetic beads during each round of click reaction-based *in vitro* selection. Conditions are indicated at the bottom of the figure.

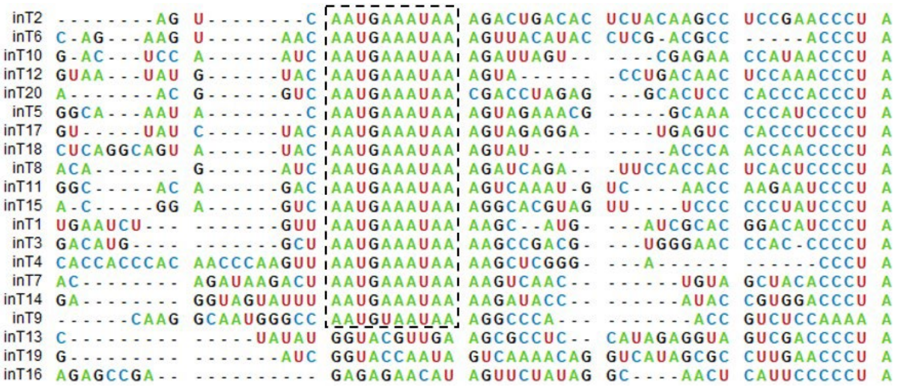

Supplementary Figure 3 | Sequence alignment of the library from the last round of click reaction-based selection. The RNA population after the last round of click reaction-based selection was sequenced and aligned. Alignment of the 40-nt anti-terminator region from the top 20 inT-boxzymes (inT1 to inT20, sorted by descending reads) was shown. A consensus motif, AAUGAAAUAA, was identified and framed by black dot lines.

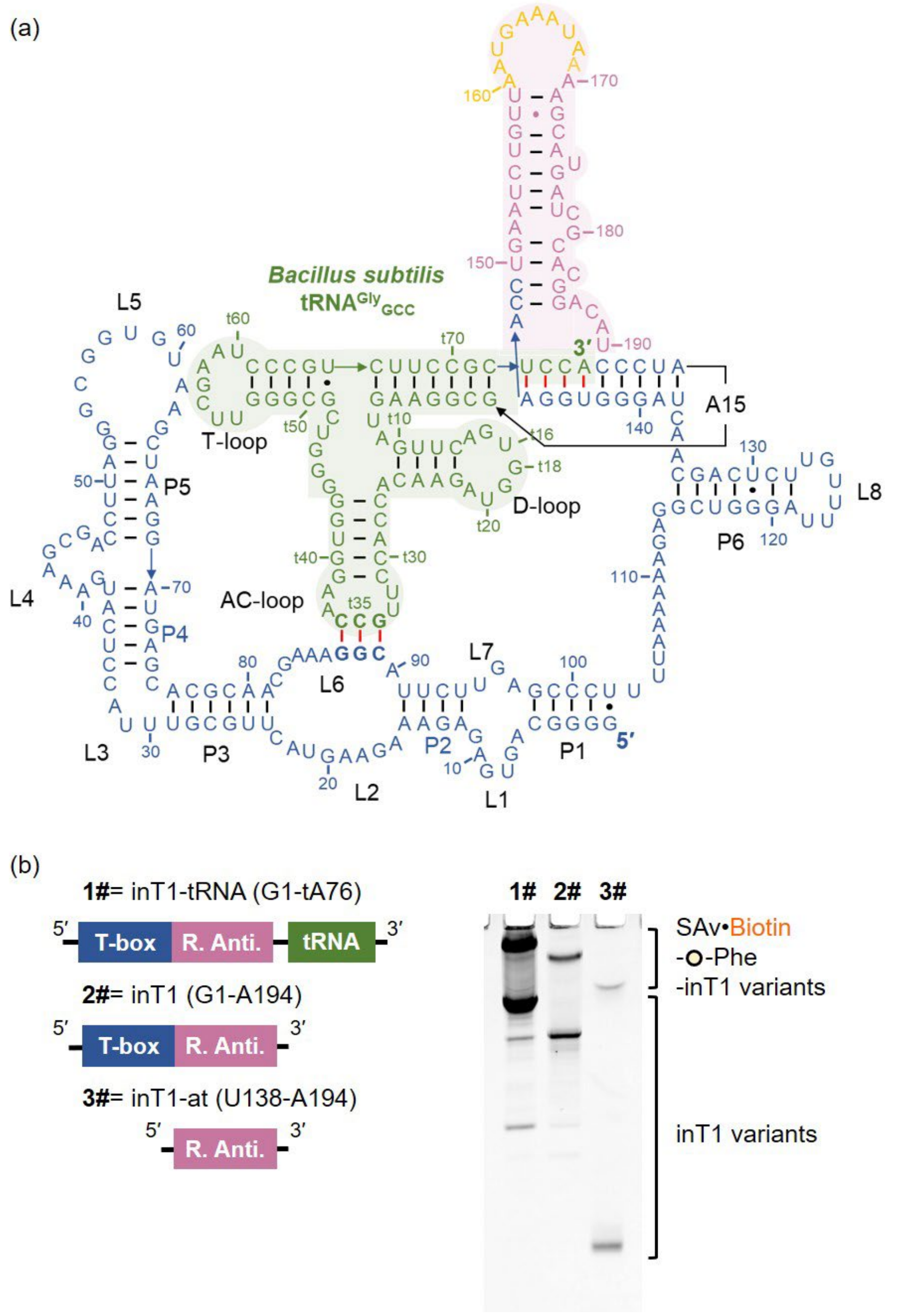

Supplementary Figure 4 | Self-aminoacylation activity of inT1-tRNA variants. (a) A predicted secondary structure of inT1-tRNA. Coloring and numbering follow Fig. S1; the consensus motif is in yellow. **(b)** Shorter derivates were prepared to elucidate inT1-tRNA's aminoacylation site. Their self-aminoacylation activity was tested by SAv-EMSA. R. Anti., randomized anti-terminator region.

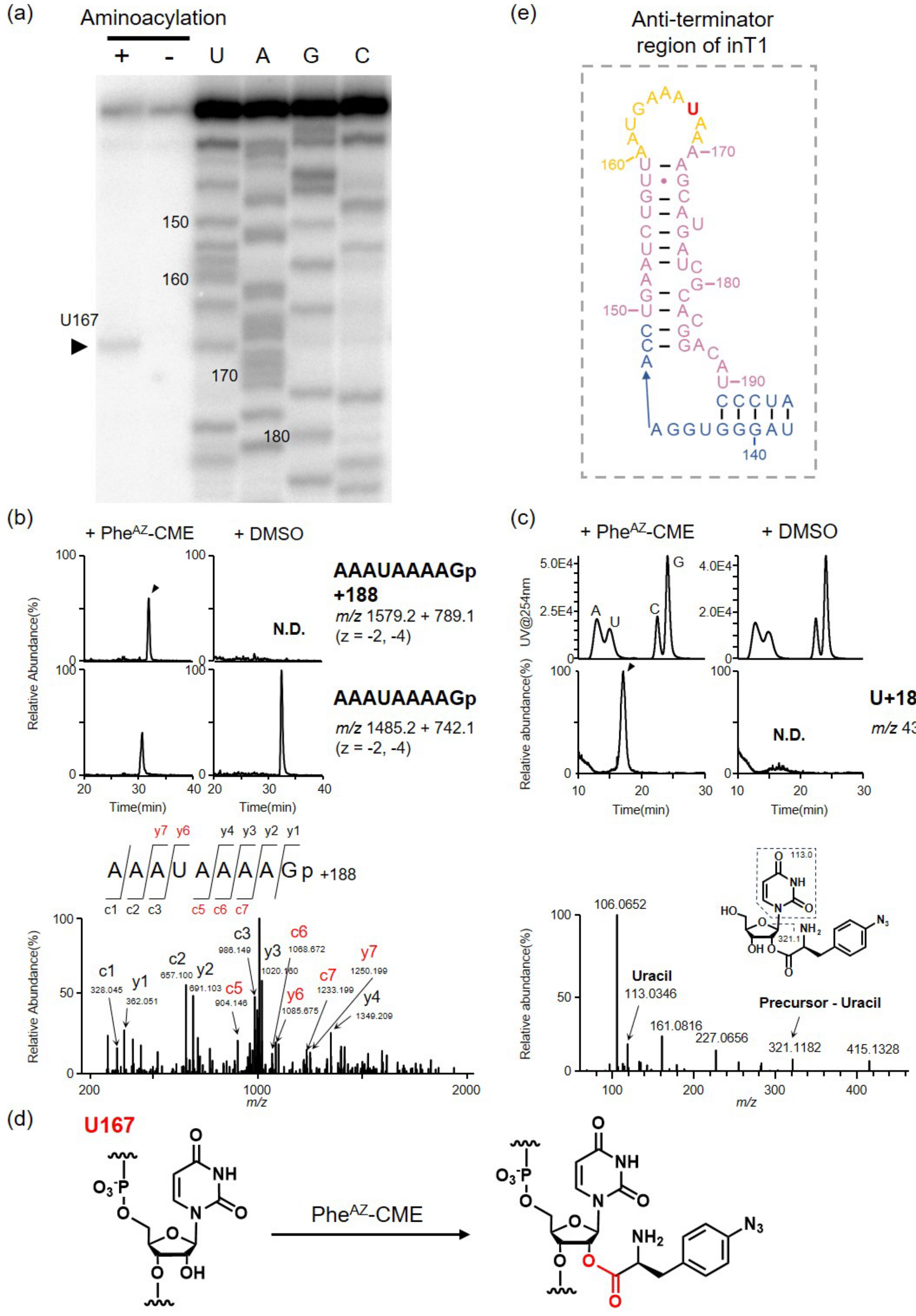

Supplementary Figure 5 | Identification of inT1 catalysis. **(a)** To determine the aminoacylation site of inT1 catalysis, we first applied primer extension assay to inT1-at-

s and identified the RT stop site, U167. Mass spectrometry analysis of inT1-at-s was also carried out. **(b)** The top figure shows the Extracted Ion Chromatogram (XIC) of RNase T_1 digested inT1-at-s fragment. The upper and lower panels show the XIC of [AAAUAAAAGp+188] and unmodified fragments. XIC peak was summed with divalent and tetravalent negative ions. N.D., non-detected. The lower figure shows the Collision-induced dissociation (CID) spectrum of the [AAAUAAAAGp+188] fragment (*m/z* 1052.64). Product ions of the c- and y-series are assigned. Given the lack of c4 and y5 ions, the ribose 2′-OH of U is assumed to be aminoacylated. Product ions containing +188 Da are shown in red. **(c)** The upper panel shows UV absorbance at 254nm. The lower panel shows the XIC of protonated [U+188] (*m/z* 433.15). (bottom) CID spectrum of [U+188]. Uracil base and uracil-detached ions are detected. **(d)** These two assays confirmed that inT1 catalyzes self-aminoacylation on U167 ribose 2′-OH. **(e)** The aminoacylation site U167 was colored in red in the inT1 anti-terminator region, which was derived from Fig. S4.

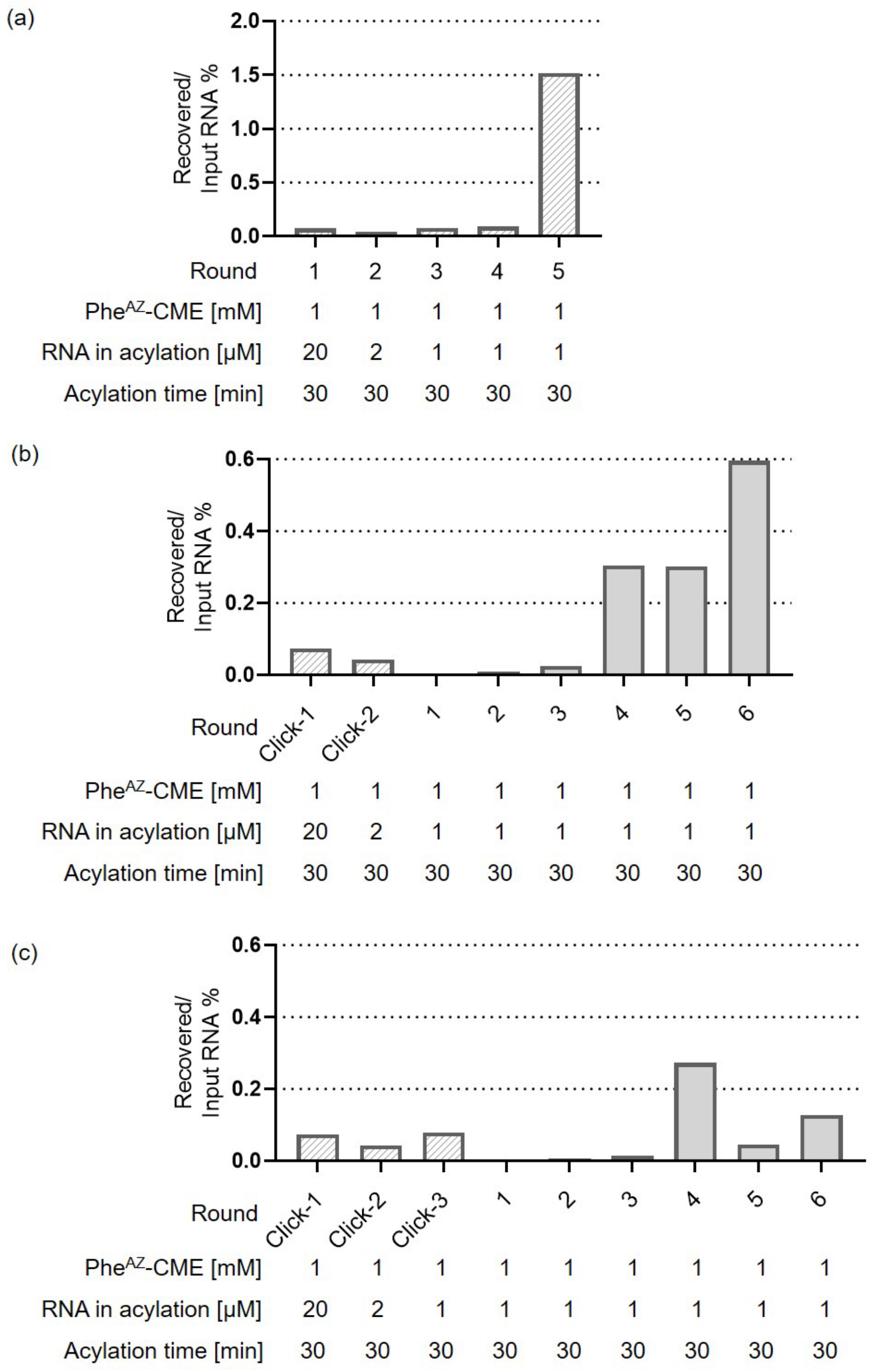

Supplementary Figure 6│The process of combined *in vitro* selection. (a) The ratio of RNA recovered from SAv-immobilized magnetic beads during each round of click-based

in vitro selection. The RNA recovery of the click-based selection increased sharply in the fifth round. We then used the RNA population after round 2 (b-tREX r2) and round 3 (b-tREX r3) of click-based selection to perform b-tREX selection. Similarly, the ratio of recovered RNA from b-tREX r2 was shown in **(b)**, and that of b-tREX r3 was shown in **(c)**. Conditions are indicated at the bottom of the figure.

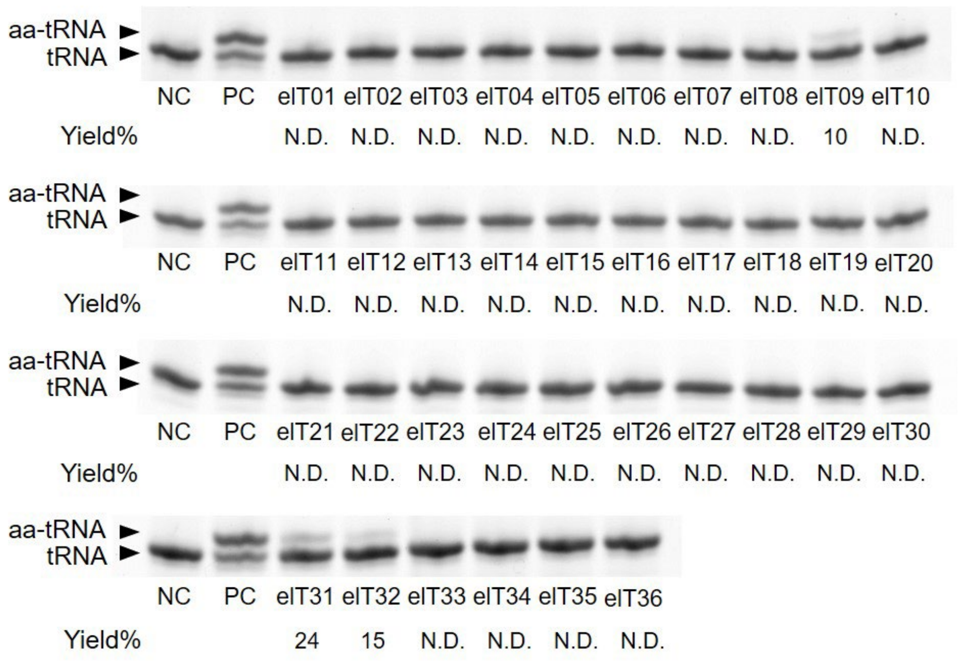

Supplementary Figure 7 | *Trans*-aminoacylation activity of 36 unique elT-boxzyme candidates from the *in vitro* selection. *Trans*-aminoacylation activity of 36 elT-boxzyme candidates was tested, and the aminoacylation yield was shown below the figure. Positive control (PC) was performed with flexizyme (eFx) (26) in the presence of 5 mM Phe^{AZ}-CME. Negative control was performed with eFx in the absence of Phe^{AZ}-CME. Among all candidates, elT09, elT31, and elT32 showed *trans*-active tRNA aminoacylation. aa-tRNA, aminoacylated tRNA. N.D., non-detected.

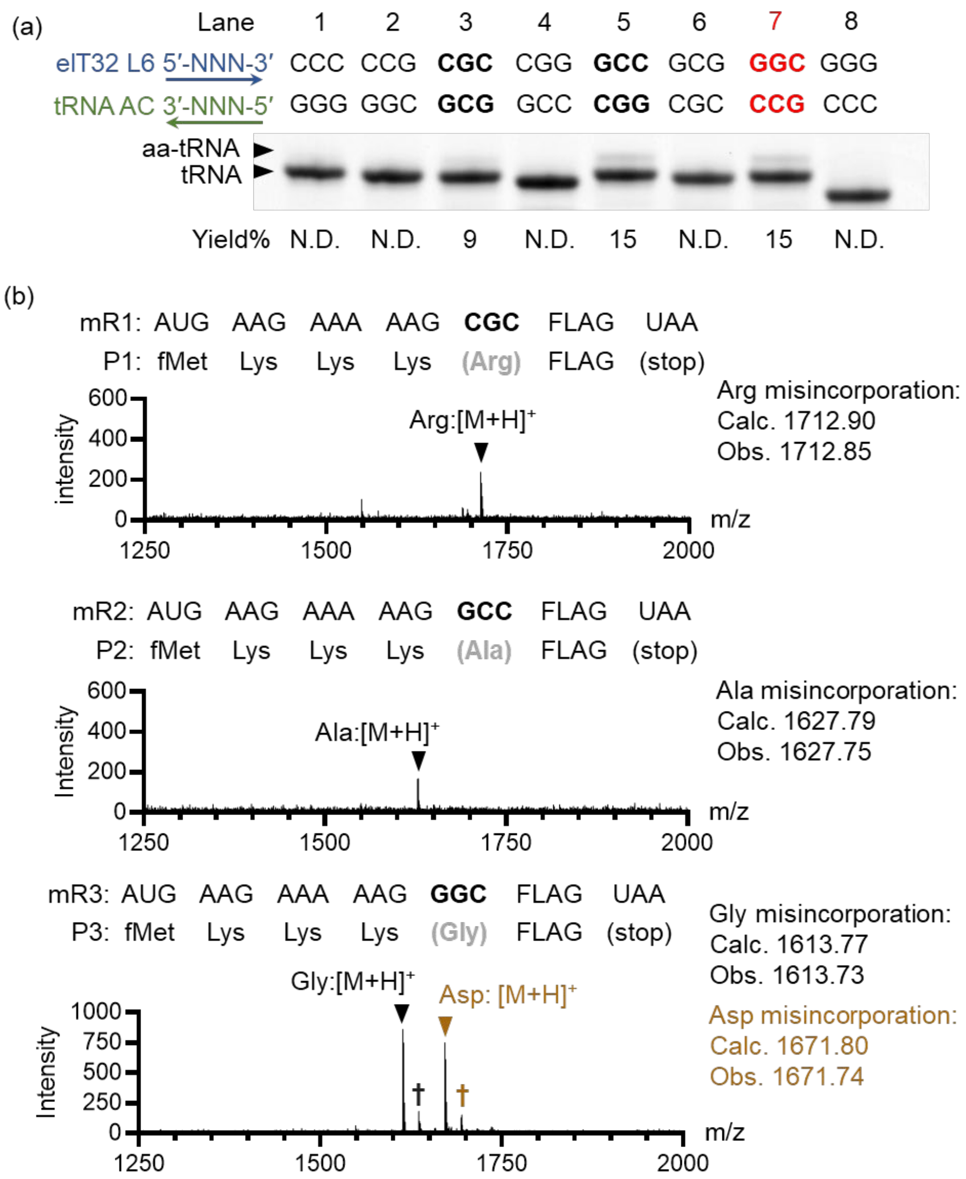

Supplementary Figure 8 | Codon optimization for lower background misincorporations. (a) eIT32 specifier sequences and cognate tRNA anticodon were flipped into GC-containing pairs. Eight combinations were tested for their *trans*-aminoacylation activity. The original GGC-GCC (Gly) pair was colored in red. aa-tRNA, aminoacylated tRNA. N.D., non-detected. **(b)** To investigate the background misincorporation, mRNA templates bearing either CGC (Arg), GCC (Ala), or GGC (Gly) elongation codons were *in vitro* translated with limited amino acids (Met, Lys, Asp, Tyr). The translation product was analyzed by MALDI-TOF MS. Calc., calculated. Obs., observed. †, the peaks corresponding to the sodium adducts of the peptide.

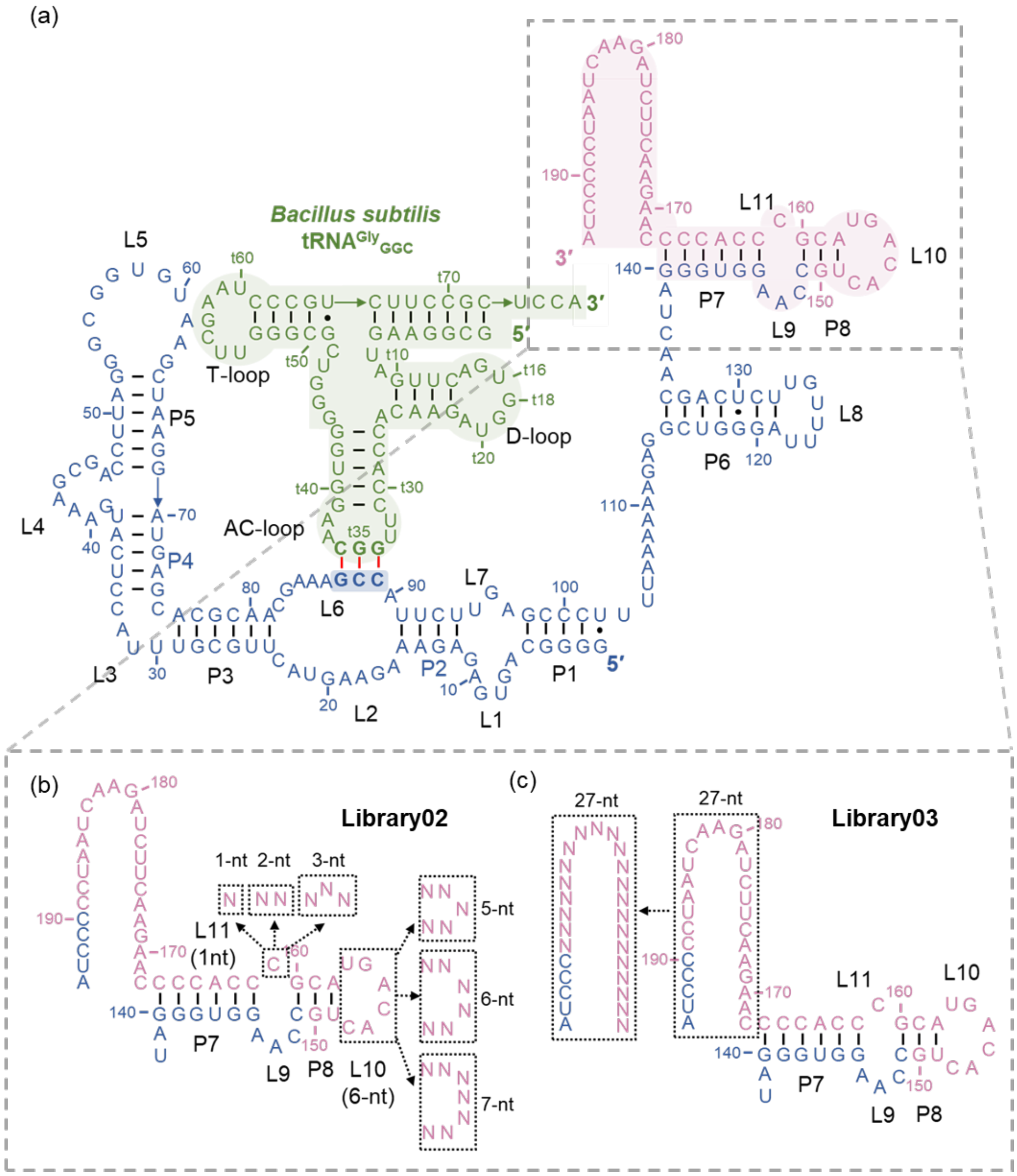

Supplementary Figure 9 | Library design for elT32 evolution. (a) A predicted secondary structure of elT32$_{GCC}$ and its unbound tRNA. Coloring and numbering followed Fig. S1. **(b)** To design a library for elT32 evolution, we focused on the predicted loops inside the anti-terminator region, namely L10 and L11. Both loops were randomized into varied loop sizes. This resulted in Library02, a combination of nine different libraries. **(c)** The last 27-nt of the anti-terminator region in elT32, predicted to be flexible, was fully randomized and resulted in Library03.

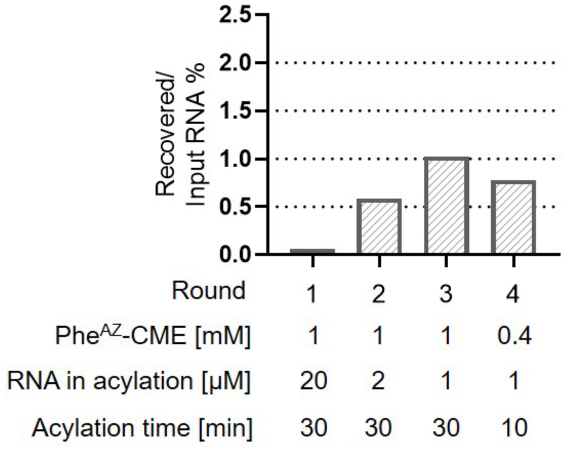

Supplementary Figure 10│The process of *in vitro* selection for elT32 evolution. The ratio of RNA recovered from SAv-immobilized magnetic beads during each round of click reaction-based *in vitro* selection for elT32 evolution. Conditions used in each round were indicated at the bottom of the figure.

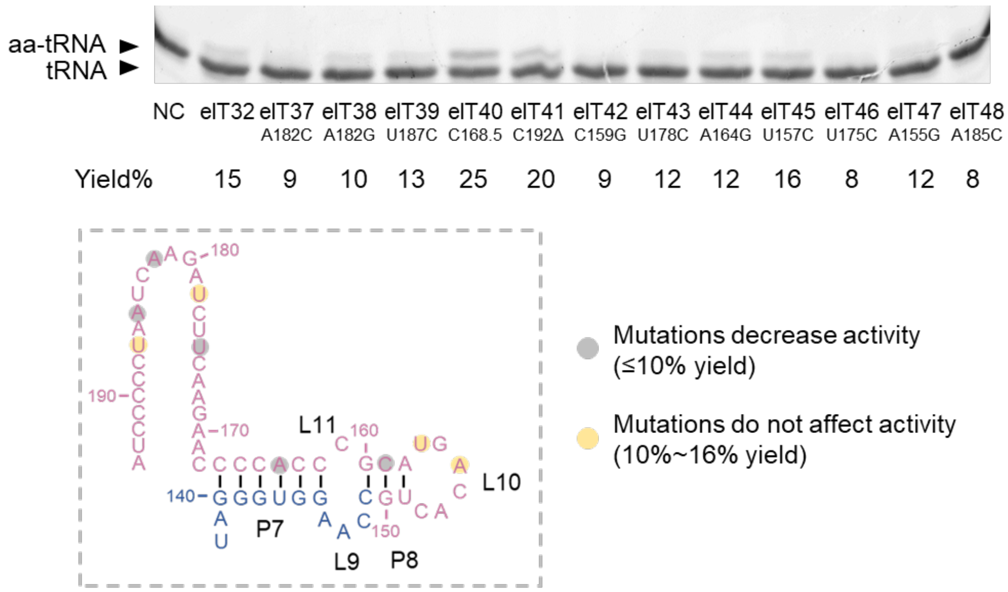

Supplementary Figure 11 | *Trans*-aminoacylation activity of elT32-mutant elT-boxzymes from the evolution. *Trans*-aminoacylation activity of 12 elT32 variants with a single mutation was tested. Negative control (NC) was performed with elT32 without Phe^{AZ}-CME. Other than elT40_C168.5 and elT41_ C192Δ with enhanced activity, ten mutants were labeled on a predicted secondary structure of the anti-terminator region of elT32, referring to their effect on the activity. elT44 (A164G) kept the activity even though the mutation was in predicted elT32's P8 stem, probably because of forming G·U wobble base pair. aa-tRNA, aminoacylated tRNA.

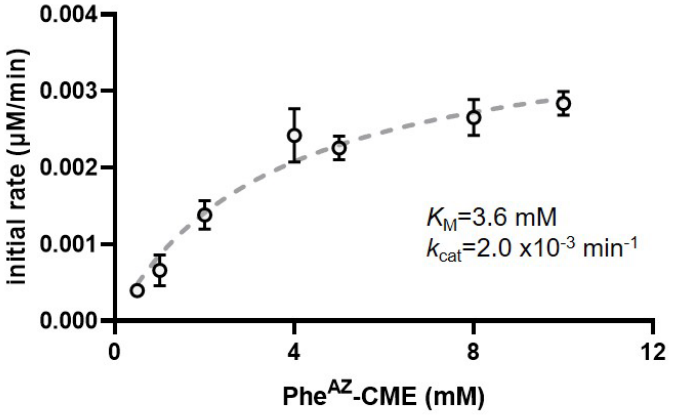

Supplementary Figure 12│Characterization of enzyme kinetics of elT49. The initial *trans*-aminoacylation rate of elT49 under 0.5-10 mM Phe^{AZ}-CME was measured three times and then fitted to the Michaelis-Menten non-linear curve.

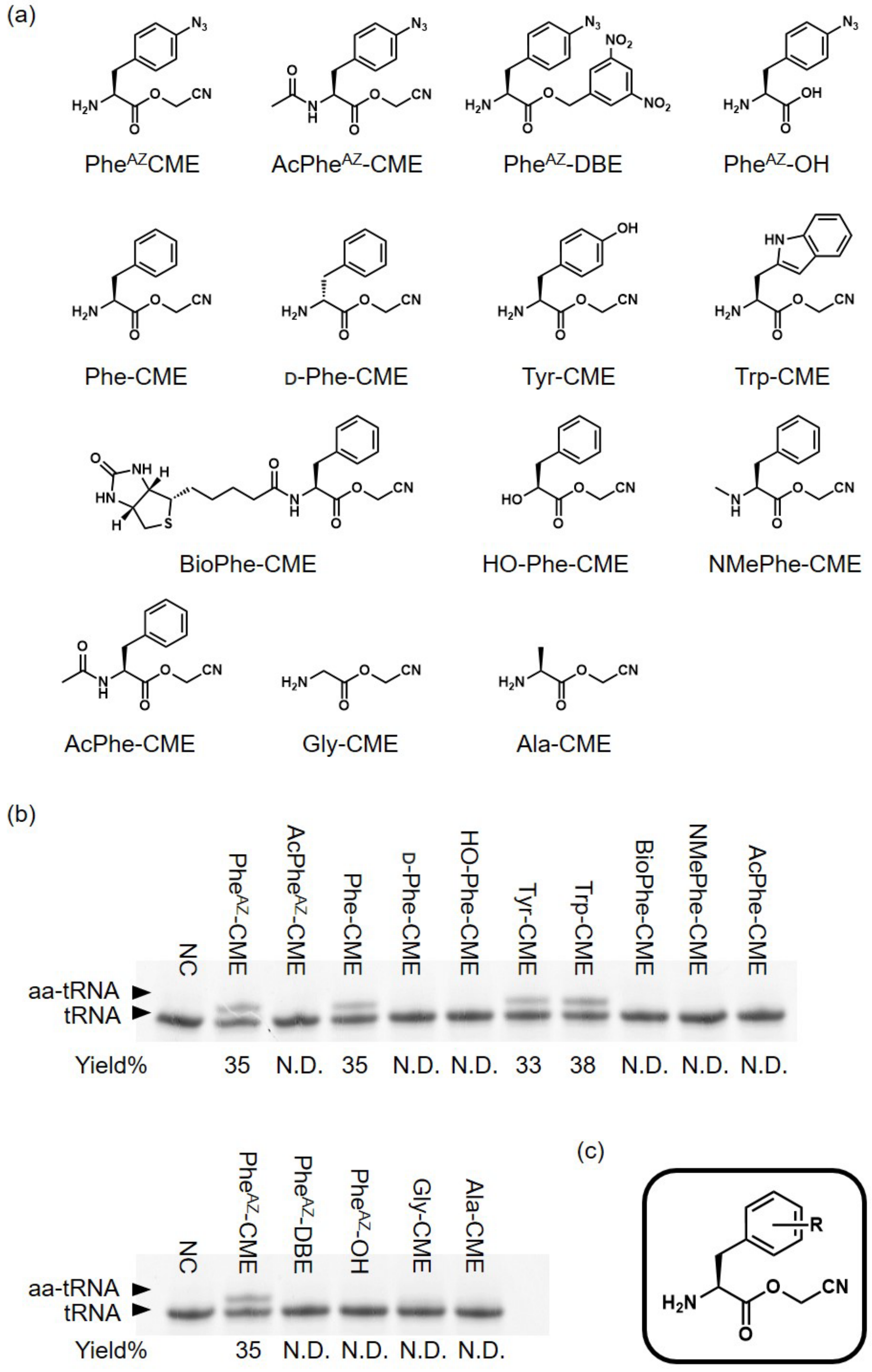

Supplementary Figure 13 | Amino acid substrates scope of eIT49. (a) Chemical structure of amino acid substrates used in this study. **(b)** eIT49 was tested for its tRNA *trans*-aminoacylation activity with all amino acid substrates shown in (a). Negative

control was performed with elT49 without any amino acid substrates. aa-tRNA, aminoacylated tRNA. N.D., non-detected. **(c)** By summarizing the entries that gave an aminoacylated-tRNA band shift, elT49 favored L-amino acids having *N*-amino, aromatic sidechain, and a CME activation group.

Reference

26. Murakami, H., Ohta, A., Ashigai, H. and Suga, H. (2006) A highly flexible tRNA acylation method for non-natural polypeptide synthesis. *Nature Methods*, **3**, 357-359.

50. Ishida, S., Terasaka, N., Katoh, T. and Suga, H. (2020) An aminoacylation ribozyme evolved from a natural tRNA-sensing T-box riboswitch. *Nature Chemical Biology*, **16**, 702-709.
